# Antioxidants in Oxidative Stress and Obesity-Associated Diseases: Molecular Mechanisms and Potential Health Implications

**DOI:** 10.3390/biomedicines14092049

**Published:** 2026-09-11

**Authors:** Bee Ling Tan

**Affiliations:** Department of Diagnostic and Allied Health Science, Faculty of Health and Life Sciences, Management and Science University (MSU), University Drive, Off Persiaran Olahraga, Seksyen 13, Shah Alam 40100, Selangor, Malaysia; tbeeling@msu.edu.my

**Keywords:** antioxidant, diabetes, flavonoid, inflammation, insulin resistance, metabolic disorder, reactive oxygen species, obesity, oxidative stress, polyphenol

## Abstract

Obesity has become a major global public health concern, with prevalence rates rising dramatically over the past several decades and affecting more than one billion people worldwide. The increasing burden of obesity has been largely driven by sedentary lifestyles and the consumption of energy-dense, nutrient-poor diets, although its etiology is multifactorial, involving complex interactions among genetic, metabolic, endocrine, and environmental factors. Beyond excess adiposity, obesity is closely associated with numerous chronic diseases, including cardiovascular disease (CVD), type 2 diabetes mellitus (T2DM), hypertension, cancer, and chronic inflammatory disorders. Emerging evidence indicates that oxidative stress plays a pivotal role in the pathogenesis of obesity and its related metabolic complications. Reactive oxygen species (ROS) and reactive nitrogen species (RNS), which are generated during normal cellular metabolism, serve important physiological functions in cell signaling and redox regulation. However, excessive production of these reactive species or impairment of endogenous antioxidant defense systems disrupts redox homeostasis, leading to oxidative damage to lipids, proteins, and nucleic acids. Such alterations contribute to cellular dysfunction, chronic inflammation, and disease progression. In this context, dietary antioxidants have attracted considerable attention due to their ability to neutralize free radicals, inhibit lipid peroxidation, and restore redox balance. Natural antioxidants derived from fruits, vegetables, and other plant-based foods may act individually or synergistically to enhance cellular defense mechanisms against oxidative stress. Furthermore, growing evidence suggests that antioxidant-rich dietary patterns may offer protective effects against obesity-associated metabolic disturbances and chronic diseases. However, clinical benefits of isolated antioxidant supplementation remain inconsistent and appear to depend on dose, bioavailability, baseline redox status, disease stage, and the preservation of physiological redox signaling. Understanding the molecular mechanisms underlying antioxidant-mediated regulation of redox homeostasis may facilitate the development of nutritional strategies for obesity prevention and management, while contributing to the reduction in oxidative stress and obesity-related disease burden and the promotion of long-term health.

## 1. Introduction

Obesity has emerged as one of the most significant global public health challenges, with its prevalence nearly tripling between 1975 and 2016 [1]. This alarming increase has been largely attributed to sedentary lifestyles and the widespread consumption of energy-dense, nutrient-poor diets characterized by high levels of refined carbohydrates and added sugars. However, the etiology of obesity is multifactorial, involving complex interactions among environmental, metabolic, endocrine, and genetic factors [2,3]. Globally, obesity prevalence has increased by approximately two percentage points per decade over recent decades [4]. According to the World Health Organization [5], more than 1 billion individuals worldwide are living with obesity, including approximately 39 million children, 340 million adolescents, and 650 million adults. Furthermore, projections indicate that by 2025, an additional 167 million children and adults may experience adverse health consequences associated with overweight and obesity [5]. Excess adiposity substantially increases the risk of numerous chronic diseases, including cardiovascular disease (CVD), hypertension, coronary heart disease, T2DM, stroke, several forms of cancer, and chronic inflammatory disorders, thereby imposing a considerable burden on global healthcare systems and reducing overall quality of life.

Oxidative stress is recognized as a key contributor to the pathogenesis and progression of numerous human diseases, including obesity, CVD, neurodegenerative disorders, and cancer [6]. Reactive oxygen species (ROS) and reactive nitrogen species (RNS) are continuously generated as by-products of normal cellular processes, particularly mitochondrial respiration, oxidative metabolism, and immune responses [7]. Under physiological conditions, these reactive species serve important roles in cellular signaling and redox homeostasis. However, excessive production or impaired antioxidant defense mechanisms can disrupt redox balance, leading to oxidative and nitrosative stress [8]. Mitochondria constitute an important intracellular source of ROS, together with NADPH oxidases and other enzymatic systems, with their relative contribution varying according to cell type and metabolic state [9,10,11]. Mitochondria generate adenosine triphosphate (ATP) through oxidative phosphorylation, a process that inevitably results in electron leakage and the formation of reactive intermediates [12]. ROS comprise a diverse group of oxygen-containing reactive molecules that can be broadly classified into radical ROS and non-radical oxidants. Radical ROS include species containing one or more unpaired electrons, such as the superoxide anion (O_2_•^−^) and hydroxyl radical (•OH), whereas non-radical oxidants include hydrogen peroxide (H_2_O_2_), singlet oxygen (^1^O_2_), and hypochlorous acid (HOCl) [9,10]. Although H_2_O_2_ is not a free radical, it functions as an important signaling molecule and serves as a precursor for the generation of more reactive oxidants, including •OH through Fenton chemistry [10]. In contrast, HOCl/OCl^−^ is primarily generated by myeloperoxidase in activated neutrophils and contributes to antimicrobial defense as well as oxidative tissue injury under inflammatory conditions [10]. Lipid peroxides are generally considered secondary products of lipid oxidation and may further propagate oxidative damage through the formation of reactive aldehydes and other downstream oxidizing species [9]. ROS play dual roles in regulating cellular proliferation, differentiation, survival, and apoptosis [13]. Nevertheless, when present at excessive levels, these reactive species can interact with proteins, enzymes, membrane lipids, nucleic acids, and other biomolecules, resulting in structural and functional alterations that contribute to cellular dysfunction and disease development.

Natural antioxidants play a critical role in protecting cells against oxidative damage by modulating redox signaling pathways, inhibiting oxidative reactions, suppressing lipid peroxidation, and improving endogenous antioxidant defense mechanisms [14,15]. Antioxidants may act individually or synergistically to neutralize excessive reactive oxygen and nitrogen species generated during cellular metabolism, thereby contributing to the maintenance of redox homeostasis and cellular integrity [16,17]. Under physiological conditions, a tightly regulated balance between pro-oxidants and antioxidants is essential for normal cellular function and survival. The interaction between redox-regulatory networks and antioxidant defense systems is highly complex and influenced by genetic, environmental, and metabolic factors. Dysregulation of redox-sensitive signaling pathways or impairment of endogenous antioxidant systems may disrupt redox equilibrium, resulting in increased susceptibility to oxidative damage and disease progression [15,18]. Given the central role of oxidative stress in aging and the development of chronic diseases, increasing attention has been directed toward dietary strategies that enhance antioxidant capacity. In particular, foods rich in natural antioxidant compounds have attracted considerable interest as nutritional interventions due to their ability to support endogenous defense mechanisms, modulate inflammatory pathways, and reduce oxidative damage [16,19]. Therefore, a better understanding of the molecular mechanisms through which dietary antioxidants regulate redox balance may provide valuable insights into the prevention and management of oxidative stress-associated diseases.

## 2. Method

A comprehensive literature search was conducted using electronic databases including PubMed, Scopus, Web of Science, and Google Scholar. The search covered studies published from January 1998 to June 2026. The following keywords and their combinations were used: “oxidative stress”, “redox signaling”, “oxidative eustress”, “oxidative distress”, “obesity”, “adipocyte dysfunction”, “type 2 diabetes mellitus”, “cardiovascular disease”, “cancer”, “antioxidants”, “polyphenols”, “carotenoids”, “glutathione”, “vitamin C”, “coenzyme Q10”, “organosulfur compounds”, “ergothioneine”, “betalains”, and “carnosine”. Boolean operators (AND, OR) were applied to refine the search strategy. Additional relevant studies were identified by manually screening the reference lists of selected articles. Studies were selected based on their relevance to redox biology, obesity-associated oxidative stress, antioxidant mechanisms, and potential health implications.

## 3. Redox Signaling, Oxidative Eustress, and Oxidative Distress

Recent advances in redox biology have reshaped the traditional view of ROS as solely harmful metabolic byproducts. According to the contemporary framework proposed by Sies et al. [18], ROS, together with RNS and reactive sulfur species (RSS), serve as essential signaling mediators that regulate a wide range of biological processes, including cellular proliferation, differentiation, metabolism, immune responses, and adaptation to environmental stimuli. Through reversible oxidation of redox-sensitive protein residues, particularly cysteine residues, these reactive species function as molecular switches that coordinate intracellular signaling networks and maintain cellular homeostasis. Consequently, physiological ROS production is not inherently detrimental but is required for normal cell function and adaptive responses. This concept is encompassed within oxidative eustress, which describes the beneficial effects of low to moderate oxidant levels that support redox signaling and cellular resilience. In contrast, oxidative distress arises when excessive or persistent oxidant generation overwhelms endogenous antioxidant and repair systems, leading to irreversible oxidative modifications of lipids, proteins, and nucleic acids that compromise cellular integrity and function [18].

**Figure 1:**
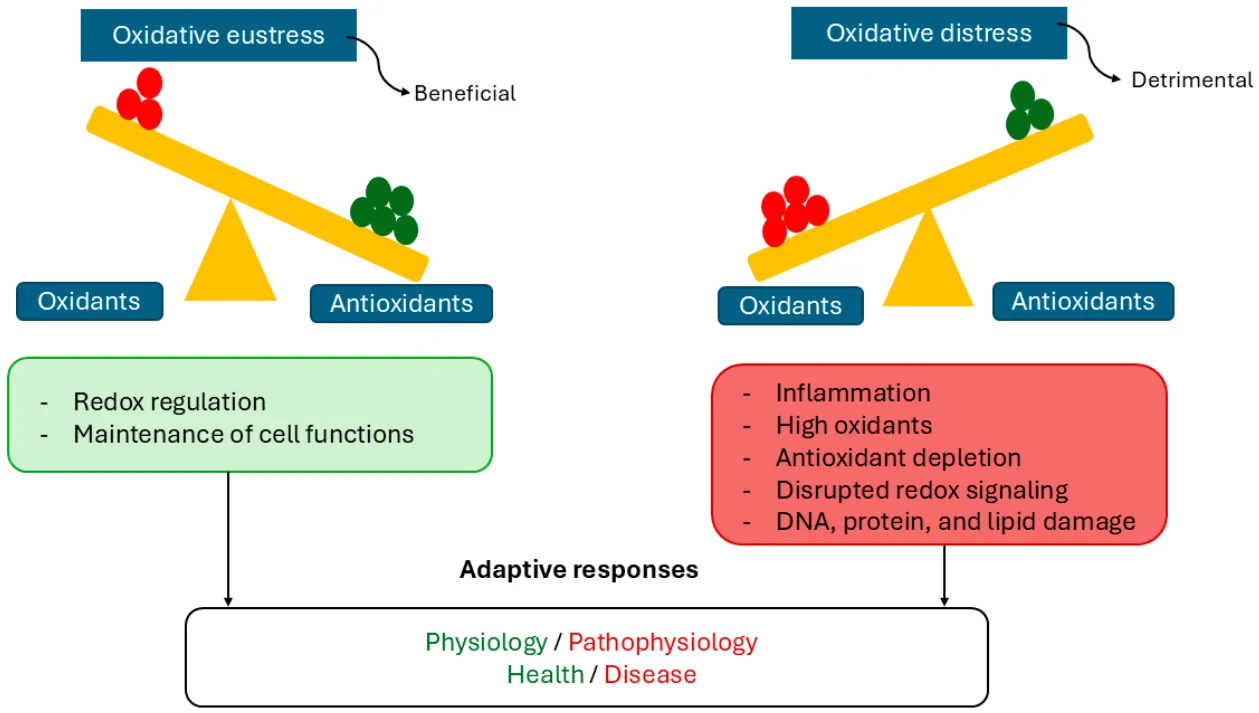
Redox signaling continuum from oxidative eustress to oxidative distress. Reactive species and antioxidant systems interact dynamically to maintain redox homeostasis. Under physiological conditions, controlled oxidant production and adequate antioxidant defenses support oxidative eustress, in which redox signaling regulates cellular processes, promotes adaptive responses, and maintains normal cell function. This beneficial state facilitates physiological adaptation and resilience through tightly regulated redox-dependent mechanisms. However, when oxidant generation exceeds antioxidant capacity, or when redox signaling becomes dysregulated, cells transition to oxidative distress. This pathological state is characterized by inflammation, excessive oxidant accumulation, antioxidant depletion, and oxidative damage to DNA, proteins, and lipids, thereby contributing to cellular dysfunction and disease development.

The distinction between oxidative eustress and oxidative distress is particularly relevant in obesity-associated diseases. Under physiological conditions, moderate ROS production contributes to cellular adaptation through hormetic responses, whereby mild oxidative stimuli activate protective pathways such as Nrf2-mediated antioxidant defenses and stress-response mechanisms. However, obesity-induced adipose tissue dysfunction, chronic inflammation, mitochondrial abnormalities, and nutrient excess can disrupt redox homeodynamics and promote excessive ROS accumulation. This shift toward oxidative distress contributes to DNA damage, genomic instability, dysregulated signaling pathways, and a pro-inflammatory microenvironment that collectively facilitate cancer initiation and progression. Importantly, the framework proposed by Sies et al. [18] also highlights that ROS possess both physiological and pathological functions, emphasizing that therapeutic interventions should aim to restore redox homeostasis rather than indiscriminately eliminate reactive species. Accordingly, while antioxidants may attenuate oxidative distress and reduce obesity-related oxidative damage, excessive suppression of physiological ROS signaling may impair adaptive cellular responses and, in certain contexts, influence the survival and treatment responsiveness of established tumor cells. Therefore, maintaining an optimal redox balance, rather than complete ROS elimination, represents a more biologically relevant strategy for diseases management [18]. Figure 1 shows that conceptual framework of redox signaling, oxidative eustress, and oxidative distress.

## 4. Molecular Mechanisms of Oxidative Stress-Induced Obesity and Diseases

### 4.1. Obesity and Adipocyte Dysfunction

Chronic low-grade inflammation is a hallmark of obesity and plays a pivotal role in the development of metabolic dysfunction. Elevated levels of inflammatory cytokines are closely associated with obesity, particularly abdominal adiposity, and are known to influence lipid metabolism and adipose tissue homeostasis [20]. The metabolic disturbances associated with excessive nutrient intake, including lipids, sugars, and fatty acids, are mediated through complex interactions between adipocytes and immune cells such as macrophages, monocytes, mast cells, and T lymphocytes residing within or infiltrating adipose tissue [21]. These interactions contribute to adipocyte dysfunction and the establishment of a chronic proinflammatory microenvironment. Furthermore, dietary fatty acids have been shown to upregulate protease-activated receptor 2 (PAR2), a signaling receptor implicated in obesity-associated inflammation [22].

In addition to metabolic disturbances, obesity promotes a pro-oxidant state through the activation of inflammatory pathways [23]. Inflammatory responses enhance the generation of ROS by activated immune cells, thereby exacerbating oxidative stress [24]. Obesity is also associated with alterations in adipose tissue T-cell populations and increased macrophage infiltration, both of which contribute to the sustained production of proinflammatory mediators [21,25]. Mature adipocytes, preadipocytes, and infiltrating immune cells collectively secrete cytokines and chemokines that perpetuate inflammatory signaling within adipose tissue [21]. Consequently, oxidative stress and inflammation establish a self-amplifying vicious cycle in which ROS stimulate the expression of proinflammatory mediators, including interleukin-6 (IL-6) and monocyte chemoattractant protein-1 (MCP-1), thereby promoting further macrophage recruitment and activation. Moreover, ROS activate the nuclear factor kappa B (NF-κB) signaling pathway, resulting in enhanced transcription of proinflammatory cytokines such as tumor necrosis factor-alpha (TNF-α) and IL-6 [26].

Adipose tissue is broadly classified into white adipose tissue (WAT) and brown adipose tissue (BAT), each possessing distinct physiological functions [27]. WAT constitutes the primary site of energy storage and is composed predominantly of adipocytes. Accumulating evidence indicates that obesity is characterized by excessive macrophage infiltration into WAT, highlighting the intimate relationship between adipose tissue expansion and chronic inflammation [21,27]. Macrophage accumulation disrupts the endocrine function of adipose tissue and stimulates the secretion of inflammatory mediators, leading to endothelial dysfunction, insulin resistance, and metabolic impairment [21]. Among these mediators, TNF-α is markedly elevated in obese adipose tissue and serves as a key regulator of inflammatory signaling [26,27]. TNF-α activates intracellular kinase complexes that promote NF-κB activation, which subsequently induces the expression of downstream proinflammatory cytokines, including IL-6 and interleukin-1β (IL-1β), thereby perpetuating adipose tissue inflammation and metabolic dysfunction [26].

Accumulating evidence indicates that obesity is associated with elevated oxidative stress, characterized by excessive ROS production and impaired antioxidant defense mechanisms [24,28]. One of the major contributors to ROS generation in obesity is NADPH oxidase, an enzyme complex that produces O_2_•^−^ as part of the innate immune response. Under physiological conditions, NADPH oxidase-derived ROS play an essential role in host defense by facilitating the elimination of invading pathogens. However, chronic activation of NADPH oxidase can result in excessive ROS production and oxidative damage [28]. Notably, advanced glycation end products (AGEs), which are generated following prolonged exposure to hyperglycemia and high-sugar diets, have been shown to activate NADPH oxidase and further exacerbate oxidative stress [29].

Obesity has also been associated with reductions in several key antioxidant enzymes, including glutathione peroxidase-1 (GPx1), superoxide dismutase (SOD), and catalase, particularly within adipose tissue [24]. Interestingly, this decline appears to be tissue-specific, as similar reductions have not been consistently observed in skeletal muscle or hepatic tissues. Although the underlying mechanisms remain incompletely understood, it has been proposed that prolonged exposure to an oxidative microenvironment within expanding adipose tissue may suppress antioxidant enzyme expression and activity. Consequently, the imbalance between ROS production and antioxidant defenses promotes oxidative damage and contributes to adipose tissue dysfunction. Collectively, obesity-induced oxidative stress, coupled with excessive consumption of high-fat and energy-dense diets, may exacerbate metabolic disturbances, inflammation, and obesity-related complications.

Oxidative stress has also been implicated in the development of insulin resistance, a key metabolic abnormality associated with obesity. However, not all individuals with obesity develop insulin resistance or T2DM, suggesting that additional genetic, environmental, and metabolic factors influence individual susceptibility to metabolic dysfunction [30]. Nevertheless, obesity remains one of the most significant global health challenges and represents a major risk factor for numerous chronic diseases, including CVD, T2DM, and several forms of cancer [31]. Emerging evidence suggests that oxidative stress serves as a common pathogenic mechanism linking obesity to these chronic conditions through its ability to impair cellular signaling, promote inflammation, and induce biomolecular damage. Therefore, understanding the role of oxidative stress in obesity-associated comorbidities is crucial for identifying effective preventive and therapeutic strategies. The following section discusses the mechanistic involvement of oxidative stress in the development and progression of CVD, T2DM, and cancer.

### 4.2. Oxidative Stress and Type 2 Diabetes Mellitus

T2DM is a chronic metabolic disorder that affected approximately 537 million adults worldwide in 2021, with projections indicating a rise to 643 million by 2030 and 783 million by 2045 [32]. Diabetes remains a major global health burden and is associated with substantial morbidity and mortality due to its numerous complications, including nephropathy, retinopathy, neuropathy, and macrovascular diseases [30,31]. The disease is characterized by impaired insulin action and progressive pancreatic β-cell dysfunction, resulting in inadequate insulin secretion and chronic hyperglycemia [30].

Oxidative stress is increasingly recognized as a central contributor to the pathogenesis and progression of T2DM [33]. Multiple risk factors, including obesity, aging, physical inactivity, and unhealthy dietary habits, promote excessive production of ROS and RNS, thereby disrupting redox homeostasis and impairing insulin signaling pathways [34]. Chronic hyperglycemia further exacerbates oxidative stress through the activation of several metabolic pathways, including the polyol pathway, protein kinase C (PKC) signaling, and AGEs formation, ultimately promoting vascular dysfunction and diabetic complications [35]. Indeed, mounting evidence suggests that oxidative stress serves as a critical mediator linking hyperglycemia to both microvascular and macrovascular complications in diabetes [36].

Insulin resistance is a hallmark of T2DM and represents a key mechanism linking obesity to metabolic dysfunction. It arises when insulin-responsive tissues exhibit diminished sensitivity to insulin, resulting in impaired glucose uptake and utilization. Defects in insulin signaling molecules, particularly insulin receptor substrates (IRS), have been implicated in the development of insulin resistance [23,37]. Experimental studies have demonstrated that disruption of IRS1 signaling induces insulin resistance, whereas abnormalities in IRS2 signaling are associated with more severe metabolic impairments, highlighting the critical roles of these signaling proteins in glucose homeostasis [38]. However, the observation that not all individuals with obesity develop insulin resistance or T2DM suggests that genetic, environmental, and metabolic factors collectively influence disease susceptibility [39,40].

Overnutrition and chronic excess energy intake are major drivers of oxidative stress in T2DM. Excessive nutrient availability enhances mitochondrial ROS production and increases RNS generation, resulting in oxidative damage to lipids, proteins, DNA, and cellular membranes. In addition to direct biomolecular damage, ROS modulate redox-sensitive transcription factors, including nuclear factor-kappa B (NF-κB), which promotes inflammatory signaling, cellular dysfunction, and apoptosis [41,42]. Furthermore, mitochondrial dysfunction, elevated intracellular calcium levels, chronic inflammation, and AGE accumulation synergistically amplify oxidative stress and contribute to the progressive deterioration of insulin signaling and pancreatic β-cell function [43].

The endogenous antioxidant system plays a crucial role in counteracting oxidative damage. Glutathione (GSH), one of the most important intracellular antioxidants, scavenges free radicals and maintains cellular redox balance. The regeneration of reduced GSH from glutathione disulfide (GSSG) requires glutathione reductase and its essential cofactor, nicotinamide adenine dinucleotide phosphate (NADPH) [43,44]. NADPH is described as a critical cofactor in cellular antioxidant systems, while GSH is essential for modulating ROS and maintaining redox homeostasis. Under hyperglycemic conditions, increased flux through the polyol pathway consumes substantial amounts of NADPH via aldose reductase-mediated conversion of glucose into sorbitol, thereby reducing NADPH availability for GSH regeneration [45,46]. Both reviews indicate that aldose reductase utilizes NADPH during the conversion of glucose into sorbitol and that NADPH depletion compromises GSH regeneration. Consequently, depletion of intracellular GSH weakens antioxidant defenses and exacerbates oxidative stress. Reduced GSH availability has been associated with the development of insulin resistance and T2DM, potentially due to impaired antioxidant capacity, increased oxidative damage, and altered protein turnover [43]. Collectively, oxidative stress, chronic inflammation, and dysregulated redox signaling are central mechanisms underlying the development and progression of insulin resistance and T2DM. Diets rich in refined carbohydrates and saturated fats further aggravate these pathological processes by promoting ROS production and inflammatory activation. Therefore, strategies aimed at restoring redox homeostasis and reducing oxidative stress may represent promising approaches for preventing or mitigating diabetes-associated metabolic dysfunction and its complications.

Pancreatic islets are highly specialized and vascularized microorgans that play a critical role in maintaining glucose homeostasis. These islets comprise several endocrine cell populations, including β cells, α cells, δ cells, pancreatic polypeptide (PP)-secreting cells, and ghrelin-producing γ cells [47]. Through extensive vascularization supplied by branches of the pancreaticoduodenal and splenic arteries, pancreatic islets continuously sense circulating nutrient levels and coordinate the release of insulin from β cells and glucagon from α cells to regulate blood glucose concentrations under both nutrient-rich and fasting conditions [48].

Emerging evidence suggests that pancreatic β-cell function is highly dependent on tightly regulated redox signaling involving ROS and RNS [49,50]. During glucose-stimulated insulin secretion, enhanced glycolytic flux increases ATP production through mitochondrial oxidative phosphorylation. This process is accompanied by electron leakage from the electron transport chain (ETC), resulting in the generation of O_2_•^−^ and other ROS [50]. While physiological levels of ROS participate in intracellular signaling associated with insulin secretion, chronic hyperglycemia promotes excessive ROS production through multiple mechanisms, including glucose autoxidation, mitochondrial dysfunction, and the formation of AGEs [43,51]. Consequently, sustained oxidative stress impairs β-cell function and contributes to the progressive deterioration of glycemic control.

Oxidative stress further exacerbates insulin resistance through oxidative modifications of proteins involved in insulin signaling. Increased protein carbonylation and nitrosylation have been reported in insulin-resistant and obesity-associated metabolic disorders, suggesting that excessive oxidative and nitrosative stress may compromise insulin receptor function and downstream signaling pathways [52]. In addition, ROS activate stress-sensitive signaling cascades and inflammatory pathways that further impair insulin responsiveness and metabolic homeostasis. Collectively, obesity-driven oxidative stress, chronic inflammation, β-cell dysfunction, and insulin resistance act synergistically to promote the onset and progression of diabetes mellitus, ultimately increasing the risk of metabolic and vascular complications [43,52].

### 4.3. Oxidative Stress and Cardiovascular Disease

CVD remains the leading cause of mortality globally, accounting for approximately 17.9 million deaths annually [53]. CVD encompasses a broad spectrum of disorders affecting the heart and blood vessels, including coronary artery disease (CAD), cerebrovascular disease, rheumatic heart disease, and peripheral vascular disease [53]. Notably, approximately one-third of CVD-related deaths occur prematurely in individuals younger than 70 years, while the majority of fatal events are attributed to myocardial infarction and stroke [53].

Oxidative stress is increasingly recognized as a central mechanism underlying the initiation and progression of CVD [54,55]. Multiple lifestyle-related factors, including physical inactivity, cigarette smoking, excessive alcohol consumption, and unhealthy dietary patterns, contribute to excessive ROS production and vascular dysfunction. In particular, Western-style diets characterized by high intakes of saturated fats, refined carbohydrates, and energy-dense foods, coupled with low dietary fiber consumption, have been consistently associated with increased cardiovascular risk [56,57]. These dietary and lifestyle factors can promote oxidative stress, endothelial dysfunction, and chronic low-grade inflammation, thereby accelerating cardiovascular pathogenesis [54,56].

Chronic inflammation is a hallmark of both obesity and CVD and serves as a critical link between metabolic dysfunction and cardiovascular complications [6,58]. Among inflammatory biomarkers, C-reactive protein (CRP) has been widely recognized as an independent predictor of cardiovascular risk and adverse cardiovascular outcomes [59]. Elevated CRP concentrations in individuals with obesity are closely associated with obesity-induced adipose tissue inflammation, which frequently accompanies adipose tissue expansion and increased macrophage infiltration into adipose depots. These activated immune cells, together with dysfunctional adipocytes, secrete proinflammatory cytokines, particularly IL-6 and TNF-α, which stimulate hepatic CRP synthesis and contribute to the development of chronic low-grade systemic inflammation [60]. Furthermore, oxidative stress and inflammation interact in a self-perpetuating cycle that contributes to vascular injury and atherosclerotic progression. Excessive ROS production reduces nitric oxide (NO) bioavailability, impairs endothelial function, and promotes the oxidation of low-density lipoprotein (LDL) particles, all of which are key events in atherogenesis. Simultaneously, inflammatory mediators further enhance ROS generation, amplifying vascular inflammation and promoting plaque formation and instability [55,61]. Consequently, the interplay between oxidative stress, chronic inflammation, and obesity-induced metabolic abnormalities represents a major pathogenic mechanism driving the development and progression of CVD [58,61].

Atherosclerosis is a chronic inflammatory disorder of the arterial wall and represents the primary pathological basis of CVD. The initiation of atherosclerosis is characterized by endothelial dysfunction, followed by the adhesion of circulating monocytes to the vascular endothelium and their subsequent migration into the subendothelial space [58]. This process is mediated by the upregulation of adhesion molecules, including vascular cell adhesion molecule-1 (VCAM-1), intercellular adhesion molecule-1 (ICAM-1), and selectins, which facilitate leukocyte recruitment to sites of vascular injury [62]. The expression of these adhesion molecules is stimulated by proinflammatory mediators such as TNF-α, interleukin-1 (IL-1), and CRP, thereby promoting endothelial activation and accelerating atherogenesis [63]. In particular, VCAM-1 plays a crucial role in the recruitment of leukocytes to developing atherosclerotic lesions, whereas ICAM-1 is produced by endothelial cells and macrophages in response to inflammatory cytokines, including TNF-α, interferon-gamma (IFN-γ), and IL-1 [62].

As atherosclerotic lesions progress, vascular smooth muscle cells (VSMCs) migrate from the medial layer of the arterial wall into the intima, where they proliferate and contribute to plaque formation [64]. This process is regulated by a complex network of growth factors and inflammatory mediators released by activated endothelial cells, macrophages, and other immune cells [65]. Importantly, VSMCs themselves are active participants in vascular inflammation through their production of chemokines such as monocyte chemoattractant protein-1 (MCP-1), a key mediator of monocyte recruitment. Experimental studies have demonstrated that deficiency of MCP-1 or its receptor, CC chemokine receptor 2 (CCR2), significantly attenuates atherosclerotic lesion development, highlighting the importance of this signaling axis in atherogenesis. Furthermore, oxidized low-density lipoprotein (ox-LDL), an early initiator of atherosclerotic plaque formation, stimulates the production of macrophage colony-stimulating factor (M-CSF) and other proatherogenic mediators that promote macrophage accumulation and foam cell formation [66].

The activation of endothelial cells, macrophages, and VSMCs results in the sustained release of cytokines, growth factors, and adhesion molecules that amplify vascular inflammation [67]. Among these mediators, IL-6 has emerged as a critical contributor to cardiovascular pathology because it increases circulating levels of fibrinogen, plasminogen activator inhibitor-1 (PAI-1), and CRP, thereby enhancing both inflammatory and prothrombotic responses [68]. Consequently, chronic inflammation not only accelerates plaque development but also increases the risk of plaque instability and thrombotic cardiovascular events.

Oxidative stress is another hallmark of CVD and plays a central role in the progression of atherosclerosis and cardiac dysfunction [61]. Increased ROS production, accompanied by impaired antioxidant defense systems, has been observed in both experimental models and patients with CVD [69]. High-fat diets are known to exacerbate oxidative stress by enhancing ROS generation while simultaneously reducing antioxidant capacity, thereby promoting endothelial dysfunction [70]. A key consequence of oxidative stress is the reduction in NO bioavailability, which impairs endothelium-dependent vasodilation and favors the production of vasoconstrictive mediators. This imbalance contributes to vascular dysfunction, atherosclerotic plaque development, and disease progression.

In addition to endothelial dysfunction, oxidative stress contributes to cardiovascular pathology through multiple interconnected mechanisms, including mitochondrial dysfunction, dysregulated calcium homeostasis, aberrant autophagy, myocardial fibrosis, increased ventricular stiffness, and lipid accumulation within cardiac tissues [70]. Both mitochondrial and extramitochondrial sources of ROS have been implicated in these pathological processes, while deficiencies in endogenous antioxidant defenses further exacerbate oxidative damage in the myocardium. Collectively, the interplay between oxidative stress, chronic inflammation, and impaired antioxidant capacity constitutes a major pathogenic mechanism driving the initiation, progression, and complications of CVD.

### 4.4. Oxidative Stress and Cancer

Cancer is a leading cause of death globally, accounting for approximately 10 million deaths in 2024, or nearly one in six deaths [71]. It comprises a heterogeneous group of diseases characterized by the uncontrolled proliferation of abnormal cells that can invade surrounding tissues and metastasize to distant organs. Carcinogenesis is a multistep process involving the progressive transformation of normal cells into malignant cells through the accumulation of genetic and epigenetic alterations [72]. Importantly, an estimated 30–50% of cancer cases may be preventable through the modification of established risk factors and the implementation of evidence-based preventive strategies.

Accumulating evidence indicates that oxidative stress plays a pivotal role in cancer initiation, promotion, and progression [27,73,74]. Obesity, a condition characterized by chronic oxidative stress and low-grade inflammation, has been increasingly recognized as an important mediator of cancer development. Experimental studies have demonstrated that adiponectin deficiency, commonly observed in obesity, exacerbates oxidative stress and promotes hepatic tumorigenesis in obese animal models of nonalcoholic steatohepatitis [75]. ROS contribute to carcinogenesis by inducing DNA damage, genomic instability, and mutations, thereby increasing susceptibility to malignant transformation during the early stages of tumor development [76]. Thus, oxidative stress represents a critical mechanistic link between obesity and cancer risk.

The incidence of most cancers increases with age, reflecting the cumulative effects of genetic alterations, environmental exposures, and age-related biological changes over time [77]. ROS are considered a hallmark of many malignancies and are produced by both tumor cells and infiltrating inflammatory cells within the tumor microenvironment [78]. Activated macrophages, neutrophils, and NADPH oxidase systems generate substantial amounts of H_2_O_2_, •OH, and O_2_•^−^, which contribute to oxidative damage of DNA, proteins, and lipids [6]. Moreover, interactions between ROS and RNS further intensify cellular damage and genomic instability, thereby facilitating tumor initiation and progression.

Chronic inflammation is widely recognized as a hallmark of cancer and plays a central role in tumor development and progression [79]. Indeed, many malignancies arise in tissues subjected to prolonged inflammation, infection, or chronic irritation [80]. Within the tumor microenvironment, inflammatory cells orchestrate a complex network of signaling pathways that support tumor cell survival, proliferation, invasion, and metastatic dissemination [81]. Inflammatory mediators, including cytokines, chemokines, prostaglandins, and growth factors, stimulate angiogenic pathways largely through the activation of vascular endothelial growth factor (VEGF), thereby promoting tumor vascularization and growth [82].

Pathological angiogenesis constitutes a defining feature of cancer progression and is essential for sustaining tumor growth and metastasis [83]. The tumor microenvironment is characterized by extensive infiltration of immune cells, including tumor-associated macrophages (TAMs), neutrophils, dendritic cells, natural killer cells, eosinophils, mast cells, and lymphocytes [84]. These cells release a diverse array of bioactive mediators, including ROS, RNS, matrix metalloproteinases (MMPs), interferons (IFNs), interleukins (e.g., IL-1β, IL-6, and IL-8), as well as proinflammatory enzymes such as cyclooxygenase-2 (COX-2), phospholipase A_2_ (PLA_2_), and lipoxygenase-5 (LOX-5), all of which contribute to tumor progression and tissue remodeling [6,7,85].

Oxidative stress also promotes tumor growth through the activation of redox-sensitive signaling pathways. Transcription factors such as activator protein-1 (AP-1) and NF-κB are activated by ROS and regulate genes involved in cell proliferation, survival, inflammation, and angiogenesis [86]. Activation of AP-1 can enhance cyclin D1 expression while suppressing cell-cycle inhibitory proteins, thereby promoting uncontrolled cell proliferation. Similarly, various growth factors, including insulin-like growth factor-1 (IGF-1) and fibroblast growth factor-2 (FGF-2), stimulate ROS generation in cancer cells, further enhancing tumor growth and survival signaling pathways [87].

Cancer cells further exploit antioxidant and metabolic pathways to maintain redox balance and support their survival. The oxidative pentose phosphate pathway (PPP) provides NADPH required for GSH regeneration and antioxidant defense, thereby protecting cancer cells against excessive oxidative damage [88]. Because GSH plays a crucial role in detoxifying intracellular ROS, disruption of the PPP-GSH axis can sensitize tumor cells to oxidative stress-induced apoptosis. Consequently, targeting antioxidant defense mechanisms has emerged as a promising therapeutic strategy for enhancing the efficacy of anticancer treatments. Furthermore, enzymes such as thymidine phosphorylase have been shown to promote ROS generation within tumor cells, thereby modulating cancer cell proliferation and survival [89].

**Figure 2:**
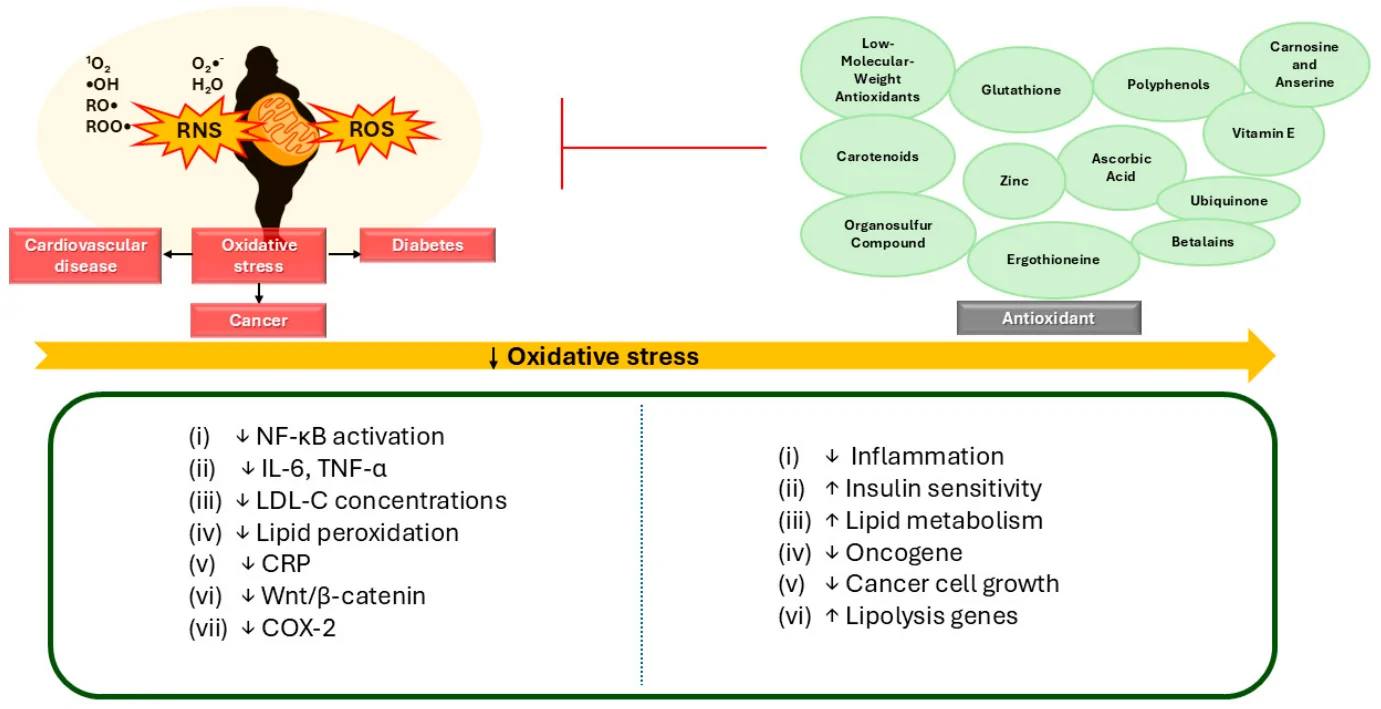
Antioxidant-mediated modulation of oxidative stress and obesity-associated diseases. Obesity promotes the generation of ROS and RNS through metabolic dysregulation, mitochondrial dysfunction, and chronic inflammation, resulting in oxidative stress and the development of obesity-related diseases, including diabetes, cardiovascular disease, and cancer. Antioxidants mitigate oxidative stress by scavenging free radicals and modulating redox-sensitive signaling pathways. These antioxidants regulate key molecular targets through suppression of NF-κB, IL-6, TNF-α, LDL-C, CRP, Wnt/β-catenin, and COX-2. These actions reduce inflammation, improve insulin sensitivity and lipid metabolism, suppress oncogenic signaling and cancer cell growth, and enhance lipolysis-related gene expression, thereby contributing to the prevention and management of obesity-associated diseases. COX-2, cyclooxygenase-2; CRP, C-reactive protein; IL-6, interleukin-6; LDL-C, low-density lipoprotein cholesterol; NF-κB, nuclear factor kappa B; Nrf2, nuclear factor erythroid 2-related factor 2; ROS, reactive oxygen species; RNS, reactive nitrogen species; TNF-α, tumor necrosis factor-alpha.

Emerging evidence suggests that hyperinsulinemia may contribute to cancer development and progression by stimulating cellular proliferation and survival pathways. For example, insulin has been identified as a growth-promoting factor in prostate cancer, and dietary strategies that reduce carbohydrate intake may lower circulating insulin concentrations and potentially attenuate tumor growth [90]. Epidemiological studies have consistently demonstrated that individuals with obesity and T2DM are at increased risk of developing several malignancies, including breast, liver, pancreatic, and colorectal cancers [91,92]. Adipose tissue expansion in obesity is associated with excessive production of ROS, resulting in systemic oxidative stress and chronic low-grade inflammation [6]. Persistent oxidative stress promotes the activation of multiple redox-sensitive signaling pathways, including NF-κB, Wnt/β-catenin, and nuclear factor erythroid 2-related factor 2 (Nrf2), which regulate genes involved in inflammation, cell proliferation, survival, and tumor progression [6]. While transient Nrf2 activation in normal cells generally exerts cytoprotective effects by enhancing antioxidant defenses and detoxification pathways; however, persistent or constitutive Nrf2 activation in established tumors may facilitate cancer cell survival, metabolic adaptation, tumor progression, and resistance to anticancer therapies [7]. Furthermore, obesity-related inflammation amplifies ROS generation, creating a self-perpetuating cycle that enhances oxidative damage and promotes a tumor-supportive microenvironment. Collectively, accumulating evidence indicates that obesity-associated adipose tissue expansion, chronic inflammation, insulin resistance, and oxidative stress act synergistically to facilitate cancer development and progression. Elevated local and systemic ROS levels derived from dysfunctional adipose tissue can exacerbate oxidative stress within the tumor microenvironment, thereby promoting genomic instability, aberrant cellular signaling, angiogenesis, and malignant progression in individuals with obesity and T2DM. Figure 2 illustrates antioxidant-mediated modulation of oxidative stress and obesity-associated diseases.

## 5. Mechanism of Action of Antioxidants

Antioxidants protect biological systems against oxidative damage by intervening at different stages of the free radical chain reaction, including initiation, propagation, and termination [93,94]. Lipid peroxidation, one of the most detrimental consequences of oxidative stress, is initiated when ROS attack polyunsaturated fatty acids within cellular membranes, resulting in the formation of lipid radicals. This process can be triggered by a variety of endogenous and exogenous factors, including mitochondrial ETC dysfunction, NADPH oxidase activation, xanthine oxidase (XO) activity, uncoupled nitric oxide synthase, cytochrome P450 enzymes, ionizing radiation, ultraviolet (UV) exposure, cigarette smoking, and environmental pollutants [94,95]. Once initiated, lipid peroxidation propagates through a chain reaction that amplifies oxidative damage and disrupts membrane integrity [96,97].

During the propagation phase, molecular oxygen rapidly reacts with carbon-centered lipid radicals to generate lipid peroxyl radicals (ROO•), which subsequently abstract hydrogen atoms from neighbouring lipid molecules and perpetuate the oxidative chain reaction [93,96]. These highly reactive intermediates can further interact with carbon-carbon double bonds, particularly in polyunsaturated fatty acids, leading to the formation of lipid hydroperoxides, cyclic peroxides, and other secondary oxidation products that contribute to cellular dysfunction and tissue damage [96,97].

Antioxidants counteract oxidative damage through multiple complementary mechanisms. These mechanisms include direct scavenging of highly reactive free radicals, quenching of ^1^O_2_, chelation of transition metal ions, inhibition of radical generation, termination of lipid peroxidation chain reactions, and modulation of endogenous antioxidant defense systems [93,94]. Direct radical scavenging primarily involves the donation of electrons or hydrogen atoms to highly reactive species such as •OH, alkoxyl radicals (RO•), and ROO•, thereby limiting oxidative injury to lipids, proteins, and nucleic acids [93,94].

In contrast, the removal of O_2_•^−^ and H_2_O_2_ predominantly depends on endogenous enzymatic antioxidant systems. SOD catalyzes the conversion of O_2_•^−^ into H_2_O_2_, which is subsequently detoxified by catalase, GPx, and other peroxide-metabolizing enzymes to maintain cellular redox balance [98]. Furthermore, many dietary and endogenous antioxidants exert indirect antioxidant effects by enhancing cellular defense mechanisms, including the activation of redox-sensitive signaling pathways such as the Nrf2 pathway, which promotes the expression of antioxidant and detoxification enzymes [98,99]. Through the coordinated actions of direct radical scavenging, enzymatic peroxide removal, and the induction of endogenous antioxidant defenses, antioxidants contribute to the preservation of cellular redox homeostasis and protection against oxidative stress-induced damage.

## 6. The Role of Antioxidants in Oxidative Stress and Obesity-Induced Diseases

### 6.1. Low-Molecular-Weight Antioxidants

Low-molecular-weight antioxidants (LMWAs) constitute an essential component of the endogenous antioxidant defense system and include compounds such as GSH, phenolic compounds, carotenoids, vitamins C and E, lipoic acid, uric acid, and coenzyme Q [98,99]. This biologically active molecule with molecular weights below 900 Da, participates in numerous physiological processes and plays a role in maintaining cellular redox homeostasis [98,99]. LMWAs protect against oxidative damage through multiple mechanisms, including free radical scavenging, metal ion chelation, and stabilization of cellular redox balance [98]. Some antioxidants, such as vitamin C (ascorbic acid) and vitamin E (tocopherol), must be obtained from dietary sources because they cannot be synthesized by humans [9]. In contrast, several endogenous compounds, including GSH, uric acid, melatonin, taurine, lipoic acid, keto acids, coenzyme Q, and melanins, also contribute substantially to antioxidant defense [98].

The biological importance of LMWAs is particularly evident in obesity and obesity-associated metabolic disorders, which are characterized by chronic oxidative stress and impaired redox regulation. Excess adiposity promotes the overproduction of ROS through mitochondrial dysfunction, chronic low-grade inflammation, and increased activity of pro-oxidant enzymes, leading to oxidative damage of lipids, proteins, and DNA [43,100]. In response to these challenges, LMWAs serve as a first line of defense by modulating ROS and maintaining intracellular redox balance. However, persistent oxidative stress in obesity can overwhelm antioxidant defenses and contribute to depletion of endogenous antioxidant reserves.

LMWAs are also implicated in obesity-related metabolic disorders, particularly T2DM. Experimental studies have demonstrated that embryos cultured under diabetic conditions exhibit significantly reduced levels of LMWAs, suggesting increased vulnerability to oxidative stress during development [101]. Moreover, diabetes has been associated with decreased concentrations of key antioxidants, including vitamin C and vitamin E, indicating disruption of antioxidant homeostasis under hyperglycemic conditions [101]. Clinical evidence further supports this association, as patients with T2DM frequently exhibit diminished circulating antioxidant status and reduced levels of several LMWAs [43,100]. Among these molecules, GSH is one of the major intracellular low-molecular-weight thiol antioxidants and redox buffers [43,102,103]. Given its central role in counteracting obesity-associated oxidative stress and metabolic dysfunction, a detailed discussion of glutathione and its biological functions is warranted.

### 6.2. Glutathione and the Glutathione-Dependent Antioxidant System

GSH is a multifunctional tripeptide composed of glutamate, cysteine, and glycine and represents one of the most abundant intracellular antioxidants in both prokaryotic and eukaryotic organisms [103,104]. This biologically active molecule, owing to their central role in maintaining cellular redox homeostasis, are indispensable for numerous physiological processes, including antioxidant defense, detoxification, immune regulation, and cellular signaling [104,105]. The unique γ-glutamyl bond linking glutamate to cysteine confers resistance to intracellular peptidases, enabling GSH to accumulate at millimolar concentrations within cells and function as a major non-protein thiol antioxidant [104].

GSH biosynthesis occurs through two ATP-dependent enzymatic reactions catalyzed by glutamate–cysteine ligase (GCL; formerly γ-glutamylcysteine synthetase) and glutathione synthetase (GS) [104,106]. In the first and rate-limiting step, GCL catalyzes the formation of γ-glutamylcysteine from glutamate and cysteine. Subsequently, GS adds glycine to generate GSH [104]. Although GCL is generally considered the principal regulatory enzyme in GSH biosynthesis, growing evidence suggests that GS may also play important roles in maintaining GSH homeostasis under specific physiological and pathological conditions [104].

The biological functions of GSH are largely attributed to the reactive sulfhydryl (-SH) group of its cysteine residue, which enables GSH to participate in redox reactions and free radical detoxification [103,106]. GSH serves as a primary defense against ROS and RNS through both direct scavenging mechanisms and GPx-mediated reduction of peroxides [104,106]. In addition, GSH plays a role in cellular detoxification by conjugating electrophilic endogenous metabolites and xenobiotics via glutathione S-transferases (GSTs), thereby facilitating their elimination and reducing cellular toxicity [104,106].

Intracellular GSH is predominantly localized in the cytosol, which contains approximately 80–90% of total cellular GSH, whereas smaller pools are distributed within mitochondria, the endoplasmic reticulum, and the nucleus [104]. Mitochondrial GSH is particularly important because it protects mitochondrial components from oxidative damage and preserves ETC function. Depletion of mitochondrial GSH has been associated with excessive ROS generation, impaired ATP production, mitochondrial dysfunction, and increased susceptibility to apoptosis [104]. Consequently, alterations in intracellular GSH concentrations can have profound effects on cellular physiology, influencing detoxification pathways, cell proliferation, stress responses, and programmed cell death [104].

Numerous studies have demonstrated that elevated GSH synthesis is a common adaptive response to oxidative stress, whereas GSH depletion is frequently associated with the onset and progression of chronic diseases, including metabolic disorders, neurodegenerative diseases, CVD, and cancer [105,106]. During conditions of increased ROS production, such as inflammation, immune activation, and enhanced metabolic activity, cells upregulate pathways involved in GSH biosynthesis and recycling to restore redox balance and prevent oxidative damage [43,105]. Therefore, maintaining adequate intracellular GSH levels is essential for modulating cellular homeostasis and regulating oxidative stress-induced injury.

A key regulator of GSH homeostasis is the Nrf2 signaling pathway, which serves as a master regulator of cellular antioxidant defenses [43,106]. Under basal conditions, Nrf2 is sequestered in the cytoplasm by Kelch-like ECH-associated protein 1 (Keap1), which targets Nrf2 for ubiquitination and proteasomal degradation. During oxidative or electrophilic stress, conformational modifications of Keap1 facilitate the dissociation of Nrf2, allowing its translocation into the nucleus [43]. Once activated, Nrf2 binds to antioxidant response elements (AREs) in the promoter regions of target genes, inducing the expression of numerous antioxidant and phase II detoxification enzymes, including those involved in GSH synthesis, regeneration, and utilization. Consequently, activation of the Nrf2–ARE pathway enhances intracellular GSH availability, reinforces antioxidant defenses, and contributes to the maintenance of cellular redox homeostasis [43].

Mounting evidence indicates that GSH plays a role in the pathogenesis and progression of T2DM. Lutchmansingh et al. [107] demonstrated that patients with T2DM exhibit significantly lower erythrocyte GSH concentrations and reduced rates of GSH synthesis compared with healthy individuals, with the greatest reductions observed among patients with microvascular complications. These findings suggest that impaired GSH homeostasis contributes to enhanced oxidative stress and the progression of diabetes-related complications. Indeed, T2DM is characterized by chronic hyperglycemia, excessive ROS production, and elevated circulating proinflammatory cytokines, all of which increase oxidative burden and accelerate GSH depletion [43]. Furthermore, activation of the polyol pathway under hyperglycemic conditions consumes NADPH, thereby limiting the regeneration of reduced GSH and further compromising cellular antioxidant defenses [43,107].

Beyond changes in GSH concentrations, alterations in GSH-dependent enzymes also contribute to oxidative stress-related disease progression. Among these enzymes, GPx plays a critical role in utilizing GSH to detoxify H_2_O_2_ and lipid hydroperoxides, thereby linking GSH availability to antioxidant function. Alterations in the GSH-dependent antioxidant system are also evident in patients with impaired glucose metabolism. For example, erythrocyte GPx activity has been reported to be significantly elevated in individuals with prediabetes and T2DM compared with normoglycemic controls [108]. Increased GPx activity appears to represent a compensatory adaptive response to heightened oxidative stress and lipid peroxidation associated with poor glycemic control. As a selenium-dependent antioxidant enzyme, GPx catalyzes the reduction of H_2_O_2_ and lipid hydroperoxides using GSH as a reducing substrate, thereby limiting oxidative damage and preserving cellular integrity [43]. Consistent with this protective role, GPx activity has been positively associated with glycated hemoglobin (HbA1c) levels, suggesting that progressive hyperglycemia stimulates endogenous antioxidant responses to counteract increasing oxidative stress [108].

Beyond its role in diabetes, GSH is increasingly recognized as a key determinant of cardiovascular and cardiometabolic health [106]. Alterations in GSH concentrations and redox status have been consistently observed during the development and progression of CVD [106]. Experimental evidence demonstrated that administration of liposomal GSH (50 mg/kg/day for two months) to apolipoprotein E-deficient mice reduced lipid peroxide and ox-LDL levels while increasing tissue GSH concentrations, resulting in attenuation of atherosclerotic progression [109]. These beneficial effects may partly be attributed to enhanced cholesterol catabolism and bile acid synthesis through increased activity of cholesterol 7α-hydroxylase [110,111].

Compelling clinical evidence further supports the involvement of GSH-dependent antioxidant systems in cardiovascular protection. In a prospective cohort study involving 909 patients with atrial fibrillation followed for a median of 43.4 months, lower serum GPx3 activity was associated with an increased risk of cardiovascular events [112]. Notably, GPx3 activity declined progressively with advancing age, with the most pronounced reductions observed in individuals aged 70 years and above. These observations suggest that age-related deterioration of antioxidant defenses may contribute to increased cardiovascular vulnerability in older adults [106,112].

Experimental studies have further highlighted the antithrombotic role of GPx3. Deficiency of GPx3 increases platelet activation, enhances thrombus formation, and promotes vascular occlusion compared with wild-type controls [113]. This phenomenon is likely mediated by increased accumulation of H_2_O_2_, which stimulates thromboxane A_2_ synthesis and subsequently enhances platelet aggregation [114,115]. Moreover, higher circulating GPx3 levels have been associated with reduced carotid intima-media thickness and lower carotid plaque burden, suggesting that diminished GPx3 activity may serve as an independent predictor of atherosclerotic disease in patients with T2DM [116]. Collectively, impairment of glutathione homeostasis and dysfunction of the glutathione-dependent antioxidant system represent important mechanisms linking oxidative stress to both T2DM and CVD. Reduced intracellular GSH availability compromises cellular redox balance and detoxification capacity, whereas alterations in GSH-dependent enzymes such as GPx further weaken antioxidant defenses and increase susceptibility to oxidative damage. Together, these disturbances contribute to chronic inflammation, vascular dysfunction, and metabolic abnormalities. Table 1 summarizes selected *in vitro* and *in vivo* studies evaluating antioxidants in obesity-associated diseases.

### 6.3. Polyphenols

Polyphenols, also known as polyhydroxyphenols, are a diverse group of plant-derived secondary metabolites characterized by the presence of multiple phenolic structural units [131,132]. These bioactive compounds are widely distributed in fruits, vegetables, cereals, legumes, teas, and other plant-based foods and are among the most abundant dietary antioxidants in the human diet [133]. The number, arrangement, and substitution patterns of hydroxyl groups on the aromatic rings largely determine the physicochemical properties and biological activities of polyphenols, including their antioxidant, anti-inflammatory, and cardioprotective effects [134]. In addition to their biological functions, polyphenols contribute to the color, flavor, astringency, and aroma of many plant-derived foods and are responsible for the characteristic pigmentation of flowers, fruits, and leaves [132,135].

Among the various classes of polyphenols, flavonoids represent the largest and most extensively studied group due to their broad spectrum of biological activities [136]. Structurally, flavonoids consist of two aromatic rings linked by a heterocyclic pyran ring and are further classified into flavonols, flavones, flavanones, flavan-3-ols, anthocyanins, and isoflavones. Numerous studies have demonstrated that flavonoids exert potent antioxidant effects through multiple mechanisms, including direct scavenging of ROS, metal ion chelation, modulation of antioxidant enzyme activity, and regulation of redox-sensitive signaling pathways [137,138,139]. In addition to their free radical scavenging capacity, flavonoids have been shown to inhibit several pro-oxidant enzymes involved in ROS generation, including XO, cyclooxygenase (COX), lipoxygenase (LOX), and phosphoinositide 3-kinase-associated signaling pathways [137,140]. Among flavonoids, luteolin (3′,4′,5,7-tetrahydroxyflavone) has been identified as a particularly potent inhibitor of XO, an enzyme that catalyzes purine metabolism and generates superoxide radicals and H_2_O_2_ as by-products [141,142]. By suppressing XO activity, flavonoids contribute to reducing oxidative stress and protecting cellular components from oxidative damage.

Furthermore, the antioxidant efficacy of flavonoids is strongly influenced by their capacity to chelate transition metal ions such as iron and copper, which catalyze the formation of highly reactive •OH through Fenton-type reactions [143]. Through these complementary mechanisms, polyphenols not only neutralize existing reactive species but also prevent the generation of new radicals, thereby preserving cellular redox homeostasis and reducing oxidative damage. Consequently, increasing evidence supports the role of polyphenol-rich foods as important dietary components for the prevention and management of oxidative stress-related disorders, including obesity, diabetes mellitus, CVD, and cancer. Flavonoids have attracted considerable attention due to their beneficial effects on cardiovascular health and their ability to modulate multiple cardiovascular risk factors, including platelet aggregation, endothelial dysfunction, dyslipidemia, and lipoprotein oxidation [144,145,146]. The cardioprotective properties of flavonoids are primarily attributed to their antioxidant, anti-inflammatory, lipid-lowering, and antithrombotic activities [147,148]. Mechanistically, flavonoids can reduce oxidative stress by scavenging ROS, inhibiting pro-oxidant enzymes, improving endothelial NO bioavailability, and modulating redox-sensitive signaling pathways involved in vascular homeostasis.

Epidemiological evidence consistently supports an inverse association between flavonoid consumption and CVD risk. Several large-scale cohort studies have reported that higher dietary flavonoid intake is associated with reduced all-cause mortality and lower incidence of CVD [149,150,151]. For example, the Zutphen Elderly Study, which followed 805 men aged 65–84 years for five years, demonstrated that regular consumption of flavonoid-rich foods such as tea, apples, and onions was associated with a significantly lower risk of coronary heart disease mortality [152]. Similarly, a 15-year prospective cohort study reported that individuals with a higher flavonoid intake (≥28.6 mg/day), particularly from black tea, exhibited a reduced incidence of stroke compared with those with lower consumption levels [153]. In addition to observational findings, clinical evidence suggests that flavonoids exert favorable effects on vascular function. A meta-analysis of randomized controlled trials evaluating catechin supplementation (20–300 mg/day) reported significant improvements in flow-mediated dilation and reductions in pulse wave velocity, both of which are important indicators of endothelial function and arterial stiffness [154]. Although no significant changes were observed in certain circulating endothelial biomarkers, these findings indicate that catechins may improve vascular health through mechanisms involving enhanced endothelial responsiveness and improved vascular compliance. Consistent with these observations, other human studies have demonstrated that flavonoids can positively modulate endothelial function, partly through the preservation of NO bioavailability and attenuation of oxidative stress-induced endothelial injury [155,156]. Collectively, the evidence suggests that flavonoids exert cardiovascular protective effects through multiple complementary mechanisms, including attenuation of oxidative stress, suppression of inflammation, inhibition of platelet aggregation, improvement of endothelial function, and modulation of lipid metabolism. These multifaceted biological actions suggest that flavonoid-rich foods may contribute to the prevention and management of CVD.

Accumulating evidence from epidemiological, experimental, and clinical studies suggests that flavonoids may contribute to cancer prevention and inhibit tumor progression through multiple molecular mechanisms [157,158]. During the initiation and promotion stages of carcinogenesis, flavonoids suppress the proliferation of transformed cells by reducing oxidative stress, enhancing DNA repair mechanisms, promoting carcinogen detoxification, and inhibiting pathways involved in cell survival and proliferation [156,159]. These actions help preserve genomic stability and reduce the likelihood of malignant transformation. During the progression stage, flavonoids exhibit a broad range of anticancer activities, including antioxidant, antiproliferative, antiangiogenic, and pro-apoptotic effects [160,161,162]. A key mechanism involves modulation of xenobiotic metabolism. Flavonoids inhibit phase I enzymes, particularly cytochrome P450 isoforms such as CYP1A1 and CYP1A2, thereby reducing the metabolic activation of procarcinogens into reactive intermediates capable of damaging cellular DNA [163]. Simultaneously, flavonoids enhance the expression and activity of phase II detoxification enzymes, including GST and UDP-glucuronosyltransferase (UGT), which facilitate the conjugation and elimination of potentially harmful compounds [164,165]. Through these complementary actions, flavonoids strengthen cellular defense systems against carcinogenic insults.

In addition to their effects on detoxification pathways, flavonoids exert potent anti-inflammatory activities that contribute to their chemopreventive potential. Experimental evidence demonstrated that supplementation with a flavonoid-rich bergamot extract (70 mg/kg body weight for 12 weeks) significantly downregulated inflammatory mediators, including IL-1β, IL-6, inducible nitric oxide synthase (iNOS), COX-2, and arginase-1, while simultaneously upregulating the tumor suppressor protein p53 [117]. These findings suggest that flavonoids can suppress tumor-promoting inflammation and restore cellular mechanisms involved in growth regulation and apoptosis. Emerging evidence also indicates that flavonoids modulate multiple redox-sensitive signaling pathways implicated in cancer development. By regulating ROS accumulation, flavonoids can attenuate the activation of transcription factors such as NF-κB, AP-1, and Wnt/β-catenin signaling pathways, all of which regulate genes involved in inflammation, angiogenesis, proliferation, and metastasis. Furthermore, the antioxidant properties of flavonoids may limit oxidative DNA damage, genomic instability, and mutagenesis, thereby reducing the likelihood of tumor initiation and progression. Collectively, flavonoids exert anticancer effects through a multifaceted network of mechanisms involving the attenuation of oxidative stress, modulation of inflammatory responses, regulation of carcinogen metabolism, enhancement of cellular antioxidant defenses, suppression of angiogenesis, and induction of apoptosis. Table 2 summarizes some of the clinical trials of antioxidants in managing obesity-associated diseases and the failure of clinical trials involving antioxidants.

### 6.4. Carotenoids

Carotenoids are naturally occurring lipophilic pigments belonging to the tetraterpenoid family and are responsible for the yellow, orange, and red coloration observed in many fruits, vegetables, algae, and other biological systems [205]. These compounds absorb visible light within the wavelength range of approximately 400–550 nm, a characteristic that underlies their pigmentation properties and biological functions [205]. Carotenoids are widely distributed across nature and are synthesized by plants, algae, photosynthetic bacteria, fungi, archaea, and some animal-associated organisms [206,207]. In addition to their role in pigmentation, carotenoids contribute to photoprotection, antioxidant defense, and the maintenance of cellular homeostasis. Structurally, carotenoids consist of a C40 isoprenoid backbone formed by eight isoprene units linked in a highly conjugated polyene chain [205]. Their characteristic structure contains multiple conjugated double bonds, which are responsible for their light-absorbing properties and potent antioxidant activity. The extensive system of conjugated double bonds enables carotenoids to quench ^1^O_2_ and neutralize ROS, thereby protecting biological membranes and cellular components from oxidative damage.

Carotenoids are generally classified into two major groups, namely, carotenes and xanthophylls. Carotenes are hydrocarbon carotenoids that contain only carbon and hydrogen atoms, with representative members including α-carotene, β-carotene, γ-carotene, and lycopene [208]. In contrast, xanthophylls are oxygenated carotenoids that contain functional groups such as hydroxyl, carbonyl, aldehyde, epoxide, carboxyl, or furanoxide moieties. Common xanthophylls include lutein, zeaxanthin, astaxanthin, fucoxanthin, β-cryptoxanthin, and peridinin [205]. The presence of oxygen-containing functional groups influences their polarity, bioavailability, and biological activities, contributing to their diverse physiological functions. Beyond their structural diversity, carotenoids have attracted considerable attention because of their antioxidant, anti-inflammatory, and disease-preventive properties. The unique polyene structure enables carotenoids to effectively quench ^1^O_2_ and scavenge free radicals, thereby reducing oxidative stress and protecting cells against oxidative injury. Consequently, dietary carotenoids have been extensively investigated for their potential roles in preventing oxidative stress and obesity-associated chronic diseases, including CVD, diabetes mellitus, and cancer.

Carotenoids have garnered considerable scientific interest due to their potent antioxidant properties and broad range of biological activities in both humans and animals [209,210]. Epidemiological evidence consistently demonstrates the existence of an inverse association between dietary carotenoid intake and the risk of several chronic diseases, including CVD, metabolic disorders, and certain cancers [74,211]. These protective effects are attributed to the ability of carotenoids to modulate cellular signaling pathways involved in cell proliferation, differentiation, apoptosis, immune function, and oxidative stress responses [212,213].

As highly lipophilic molecules, carotenoids are preferentially localized within cellular membranes, where they protect membrane lipids and other biomolecules from oxidative damage induced by ROS [214]. Their extensive conjugated double-bond system enables efficient quenching of ^1^O_2_ and scavenging of free radicals, thereby preserving membrane integrity and maintaining cellular redox homeostasis. Beyond their antioxidant capacity, carotenoids have also been shown to influence cell signaling networks associated with inflammation, immunity, and metabolic regulation, further contributing to their health-promoting effects.

Several carotenoids possess provitamin A activity and serve as important precursors for vitamin A biosynthesis. These carotenoids contain at least one unsubstituted β-ionone ring and include β-carotene, α-carotene, γ-carotene, and β-cryptoxanthin [215]. Following absorption, these compounds can be enzymatically converted into retinal and other retinoids, which are essential for vision, immune competence, epithelial differentiation, and normal growth and development [216]. Consequently, carotenoids play a dual role in human health by acting both as potent antioxidants and as precursors of vitamin A. Emerging evidence further suggests that carotenoids may contribute to the prevention of obesity-related diseases by attenuating chronic inflammation, reducing oxidative damage, and supporting immune function. Given their potent antioxidant and anti-inflammatory properties, carotenoids have been extensively investigated for their potential to mitigate oxidative stress and obesity-associated metabolic disorders, including diabetes mellitus, CVD, and cancer.

Among dietary carotenoids, lycopene has received substantial attention because of its high ^1^O_2_ quenching capacity and potent antioxidant activity. Lycopene is abundant in tomatoes, tomato-based products, and watermelon, foods that are also considered important components of cardioprotective dietary patterns such as the Mediterranean diet [217]. Oxidative stress activates several proinflammatory signaling cascades, particularly NF-κB, leading to increased expression of cytokines such as IL-6 and TNF-α, both of which contribute to endothelial dysfunction and cardiovascular pathology [218]. Consequently, the ability of carotenoids to attenuate oxidative stress and inflammatory signaling has attracted considerable interest for use in CVD prevention. In addition to its antioxidant activity, lycopene has been shown to modulate inflammatory responses, improve endothelial function, and regulate apoptosis-related pathways [219]. Clinical studies evaluating lycopene-rich foods, including tomato products consumed at doses ranging from 70 to 400 g/day, have generally reported improvements in flow-mediated dilation together with reductions in circulating IL-6 and low-density lipoprotein cholesterol (LDL-C) concentrations [188]. These findings suggest that regular consumption of lycopene-containing foods may contribute to cardiovascular protection. Interestingly, some evidence suggests that the cardiovascular benefits of lycopene may be more closely related to its anti-inflammatory actions than its direct inhibition of LDL oxidation [220]. Owing to its highly hydrophobic nature, lycopene is predominantly localized within the hydrophobic core of lipoproteins, which may influence its interaction with circulating oxidized lipids. Nevertheless, lycopene has consistently demonstrated the ability to suppress inflammatory signaling pathways and reduce oxidative injury within vascular tissues [220].

Oxidative stress contributes to endothelial dysfunction through oxidative injury to endothelial cells and uncoupling of endothelial nitric oxide synthase (eNOS), resulting in reduced NO bioavailability and impaired vascular relaxation [221]. Lycopene has been shown to counteract these effects by modulating ROS accumulation, preventing lipid and protein oxidation, preserving mitochondrial function, and enhancing endothelium-dependent vasodilation through improved NO bioavailability [222]. In addition, watermelon-derived L-citrulline may further support endothelial function by increasing arginine availability for NO synthesis [223]. Notably, lycopene supplementation appears particularly beneficial in individuals with established CVD, as improvements in endothelial-dependent vasodilation have been reported in CVD patients but not consistently in healthy subjects, suggesting a potential role in secondary cardiovascular prevention [224].

At the molecular level, lycopene exerts anti-atherogenic effects through the modulation of vascular inflammation and immune responses. Experimental studies have demonstrated that lycopene suppresses TNF-α-induced NF-κB activation, reduces intracellular adhesion molecule-1 expression, and inhibits monocyte-endothelial cell interactions, all of which are critical events in the early stages of atherosclerosis [225]. Lycopene has also been shown to attenuate T-lymphocyte activation and decrease macrophage-derived matrix metalloproteinase secretion, thereby limiting vascular inflammation and plaque instability [219]. Furthermore, animal studies revealed that lycopene supplementation (30 mg/kg/day for eight weeks) reduced smooth muscle cell proliferation, intimal hyperplasia, and transplant vasculopathy by modulating the NO/cyclic guanosine monophosphate (cGMP) signaling pathway and suppressing Rho-associated kinase activity [123].

Beyond its vascular effects, lycopene may contribute to lipid homeostasis and cholesterol regulation. Evidence suggests that lycopene downregulates proprotein convertase subtilisin/kexin type 9 (PCSK9) expression and inhibits 3-hydroxy-3-methylglutaryl-coenzyme A (HMG-CoA) reductase activity, leading to reduced cholesterol synthesis and improved lipid metabolism [226]. These findings are particularly noteworthy because they suggest that lycopene may represent a complementary nutritional strategy for individuals with dyslipidemia or statin intolerance. Collectively, lycopene exerts multifaceted cardioprotective effects through the attenuation of oxidative stress, suppression of inflammatory signaling, improvement of endothelial function, enhancement of NO bioavailability, and regulation of lipid metabolism. In addition to its lipid-lowering and anti-atherogenic effects, lycopene has demonstrated antihypertensive potential. Mechanistically, lycopene enhances endothelial NO bioavailability, attenuates angiotensin II-induced oxidative stress, and inhibits ACE activity, thereby improving vascular function and contributing to the maintenance of blood pressure homeostasis [227].

In addition to their established roles in visual and cardiovascular health, carotenoids have attracted considerable attention for their potential anticancer properties, highlighting their broad functional significance as bioactive phytochemicals [228]. Among the various carotenoids, lycopene has been extensively investigated due to its potent antioxidant activity and its potential role in cancer prevention and therapy [229]. Epidemiological and experimental evidence suggests that higher dietary intake of lycopene and carotenoid-rich foods may be associated with a reduced risk of several types of cancer [230]. The anticancer activities of carotenoids are mediated through multiple complementary mechanisms, including modulation of phase I and phase II detoxification enzymes, suppression of chronic inflammation, regulation of angiogenesis and metastasis, induction of apoptosis, cell-cycle arrest, and modulation of growth factor-mediated signaling pathways [231]. These biological effects are closely linked to the unique polyene structure of carotenoids, which enables efficient scavenging of ROS and attenuation of oxidative stress-induced cellular damage [208]. By limiting oxidative DNA damage and preserving genomic stability, carotenoids may reduce the likelihood of malignant transformation and tumor progression.

Among carotenoids, lycopene has demonstrated particular efficacy in experimental models of carcinogenesis. Studies have shown that lycopene suppresses hepatic tumor initiation through enhancement of endogenous antioxidant defense systems, direct scavenging of free radicals, and inhibition of cytochrome P450 2E1 (CYP2E1), an enzyme involved in the generation of reactive metabolites and oxidative stress [232,233,234]. Furthermore, lycopene has been reported to activate the Nrf2 signaling pathway, a master regulator of cellular antioxidant responses. Activation of Nrf2 promotes the transcription of antioxidant and phase II detoxification enzymes, thereby enhancing cellular resistance to oxidative stress and reducing susceptibility to carcinogen-induced damage [235]. Emerging evidence also suggests that carotenoids can modulate multiple signaling pathways involved in tumor development, including mechanisms associated with inflammation, proliferation, apoptosis, and cellular survival. Through suppression of oxidative stress and inflammatory signaling, carotenoids may create a less favorable environment for tumor initiation and progression. These findings suggest that carotenoids may contribute to cancer prevention and could serve as adjuncts to conventional therapeutic strategies. Collectively, carotenoids exert anticancer effects through a multifaceted network of antioxidant, anti-inflammatory, detoxification-enhancing, and cell-regulatory mechanisms. Among all carotenoids, lycopene has shown mechanistic potential due to its ability to activate Nrf2-mediated antioxidant defenses, suppress oxidative stress, and modulate pathways involved in carcinogenesis and tumor progression. Despite extensive evidence supporting the antioxidant properties of carotenoids, clinical outcomes have not always been consistent with their expected protective effects. The most notable example is β-carotene supplementation in high-risk populations. In the Alpha-Tocopherol, Beta-Carotene Cancer Prevention (ATBC) Study involving 29,133 male smokers, supplementation with β-carotene (20 mg/day) unexpectedly increased lung cancer incidence by 18% and total mortality by 8% compared with participants who did not receive β-carotene supplementation [169]. Furthermore, the β-Carotene and Retinol Efficacy Trial (CARET) was terminated early after investigators observed a 28% increase in lung cancer incidence and a 17% increase in overall mortality among smokers and asbestos-exposed workers receiving β-carotene and retinyl palmitate supplementation [236]. One of the proposed explanations is that under conditions of high oxidative stress, such as cigarette smoke exposure, β-carotene may undergo oxidative degradation, generating oxidation products that promote rather than suppress oxidative damage, thereby exhibiting pro-oxidant effects. Moreover, this study may suggest that the biological actions of antioxidants are highly context dependent and influenced by factors such as dosage, baseline oxidative status, environmental exposures, and individual metabolic characteristics. Therefore, although carotenoids remain important dietary antioxidants and are associated with numerous health benefits when consumed as part of a balanced diet, evidence from large-scale intervention studies suggests that supplementation approach should be carefully evaluated in specific populations, and beneficial effects observed in experimental models cannot be directly extrapolated to clinical outcomes.

### 6.5. Zinc and Antioxidant Defense

Among the trace minerals, zinc has attracted considerable attention because of its critical role in glucose metabolism, insulin signaling, and antioxidant defense. Zinc is essential for insulin biosynthesis, maturation, storage, crystallization, and secretion within pancreatic β-cells [237,238,239,240,241]. Intracellular zinc transport into insulin secretory granules is mediated by zinc transporter 8 (ZnT8), and impairment of ZnT8 function has been shown to reduce insulin secretion and disrupt insulin crystallization [197]. Furthermore, zinc participates in insulin action by facilitating insulin receptor phosphorylation and enhancing glucose uptake into peripheral tissues [242].

Beyond its metabolic functions, zinc exerts potent anti-inflammatory and antioxidant effects. Experimental evidence suggests that zinc suppresses NF-κB activation, thereby reducing the production of proinflammatory cytokines such as TNF-α, IL-6, and IL-1β by macrophages and monocytes [243]. These anti-inflammatory actions may contribute to the attenuation of chronic low-grade inflammation commonly observed in obesity and metabolic disorders. In addition, zinc serves as a cofactor for numerous antioxidant enzymes and proteins involved in protecting cells against oxidative damage [243].

Clinical evidence further supports the role of zinc in metabolic health. A recent systematic review and meta-analysis reported that zinc supplementation improved insulin resistance and glycemic control among overweight and obese individuals, suggesting a beneficial role in the management of obesity-associated metabolic dysfunction [244]. However, findings regarding the association between zinc intake and T2DM risk remain inconsistent. While several studies have reported inverse associations between zinc status and diabetes risk, others have failed to demonstrate significant relationships between dietary or supplemental zinc intake and the development of T2DM [241]. These discrepancies may reflect differences in study populations, zinc status, dietary patterns, and methodological approaches. Collectively, dietary minerals play indispensable roles in regulating oxidative balance, metabolic homeostasis, and immune function. Emerging evidence suggests that adequate mineral status may help mitigate oxidative stress, inflammation, insulin resistance, and other pathological processes underlying obesity-related chronic diseases. Nevertheless, maintaining an optimal balance is essential, as both mineral deficiency and excessive intake may adversely affect health outcomes.

### 6.6. Ascorbic Acid

Ascorbic acid (AscH_2_), commonly known as vitamin C, is a water-soluble antioxidant that plays a fundamental role in maintaining cellular redox homeostasis and protecting biological systems against oxidative stress [245]. Structurally, ascorbic acid is a ketolactone containing two ionizable hydroxyl groups, and under physiological conditions it predominantly exists in its monoanionic form, ascorbate (AscH^−^) [245]. The antioxidant capacity of vitamin C arises from its ability to readily donate electrons, thereby acting as a potent reducing agent. During this process, ascorbate undergoes sequential one-electron oxidation reactions to form the relatively stable ascorbate radical (Asc•^−^) and subsequently dehydroascorbic acid (DHA) [246]. Owing to resonance stabilization of its unpaired electron, the ascorbate radical exhibits low reactivity and can be efficiently recycled back to ascorbate, enhancing the effectiveness of vitamin C as a physiological antioxidant [245,247].

Vitamin C participates in numerous biological processes beyond its direct antioxidant activity. It functions as an electron donor in enzymatic reactions, a scavenger of reactive oxygen and nitrogen species, and a cofactor for several enzymes involved in collagen synthesis, carnitine biosynthesis, neurotransmitter production, and epigenetic regulation [248]. Through these diverse functions, ascorbic acid contributes to cellular metabolism, tissue integrity, immune function, and overall physiological homeostasis. The presence of multiple hydroxyl groups enables ascorbic acid to neutralize a broad range of free radicals and reactive intermediates by donating hydrogen atoms or electrons [247]. In addition, vitamin C can influence metal-catalyzed redox reactions through its interaction with transition metals such as iron and copper, thereby modulating oxidative processes within biological systems [249]. This metal-chelating capacity, together with its potent free radical scavenging properties, makes ascorbic acid one of the most important water-soluble antioxidants in human tissues and extracellular fluids. Importantly, vitamin C interacts synergistically with other components of the antioxidant defense network. For example, it regenerates oxidized vitamin E (α-tocopherol) within cellular membranes and supports the maintenance of intracellular GSH levels, thereby enhancing overall antioxidant capacity and protecting biomolecules from oxidative damage [247]. Consequently, adequate vitamin C status is essential for preserving redox balance and mitigating oxidative stress-associated pathological conditions [250].

Epidemiological evidence indicates that lower plasma vitamin C concentrations are associated with elevated levels of inflammatory biomarkers, particularly CRP, suggesting a close relationship between vitamin C status, systemic inflammation, and chronic disease risk [251]. Through its ability to scavenge ROS, regenerate other antioxidants, and modulate redox-sensitive signaling pathways, vitamin C plays a crucial role in maintaining cellular homeostasis and reducing oxidative damage.

Substantial evidence also supports a protective role of vitamin C in carcinogenesis. Several epidemiological studies have reported that dietary vitamin C intake derived from fruits and vegetables is associated with a lower risk of certain cancers, including pancreatic cancer [178]. Interestingly, the protective effects of dietary vitamin C appear to be more pronounced than those observed with supplementation alone, suggesting that synergistic interactions among bioactive compounds present in whole foods may contribute to cancer prevention. Mechanistically, vitamin C exerts anticancer effects through multiple pathways, including attenuation of oxidative stress and inflammation, enhancement of DNA repair mechanisms, modulation of growth factor signaling, induction of apoptosis, regulation of cell-cycle progression, and activation of phase II detoxification enzymes [252]. These activities contribute to the maintenance of genomic stability and suppression of tumor development.

In addition to its anticancer potential, vitamin C has been extensively investigated for its cardioprotective effects. A meta-analysis of prospective cohort studies demonstrated that higher dietary intake and circulating concentrations of vitamin C were associated with a reduced risk of stroke [253]. Several biological mechanisms may underlie this association, including inhibition of lipid peroxidation, protection against oxidative modification of LDL, suppression of vascular smooth muscle cell proliferation, and preservation of endothelial function [254]. Furthermore, vitamin C has been shown to exhibit anti-inflammatory effects, which may be particularly relevant given the established role of systemic inflammation in the pathogenesis of stroke and other CVD [255,256]. By reducing oxidative stress and vascular inflammation, vitamin C may contribute to the maintenance of vascular integrity and cardiovascular health.

Evidence from population-based studies further supports the cardiovascular benefits of diets rich in fruits and vegetables. Increased consumption of fruits and vegetables has been associated with a lower risk of coronary heart disease, with each additional daily serving resulting in a measurable reduction in cardiovascular risk [257]. Although these protective effects cannot be solely attributed to vitamin C, antioxidant-rich fruits and vegetables are important contributors to overall antioxidant capacity and vascular health. Supporting this notion, Hermsdorff et al. [185] demonstrated that a diet rich in fruits and vegetables providing ≥150 mg/day vitamin C significantly increased GPx activity and total antioxidant capacity while reducing circulating oxidized LDL concentrations in healthy adults. These findings suggest that vitamin C-rich dietary patterns may enhance endogenous antioxidant defenses and protect against oxidative stress-mediated cardiovascular damage. Vitamin C may represent an important dietary factor for the prevention of obesity-associated diseases and the maintenance of long-term health.

### 6.7. Vitamin E

Vitamin E is a family of lipid-soluble, plant-derived bioactive compounds comprising eight structurally related chromanol congeners, each characterized by a hydrophobic side chain attached to the C2 position of the chromanol ring [258]. These compounds are categorized into four tocopherol (α-, β-, γ-, and δ-) and four corresponding tocotrienol isoforms, which differ in the degree of methylation of the chromanol nucleus and the saturation of their side chains [259]. Naturally abundant in lipid-rich foods such as vegetable oils, nuts, seeds, and whole grains, vitamin E may also be incorporated into fortified food products to enhance dietary intake [260]. The amphipathic structure of vitamin E facilitates its incorporation into cellular and subcellular membranes, where it functions as a critical chain-breaking antioxidant by scavenging lipid ROO• and interrupting the propagation of lipid peroxidation.

Vitamin E exhibits potent anti-atherosclerotic properties through its ability to modulate oxidative stress, inflammatory signaling, and vascular homeostasis [261]. Beyond its role as a lipid-soluble antioxidant, vitamin E regulates the activity of several signaling molecules, particularly PKC, thereby influencing endothelial function, vascular smooth muscle cell proliferation, and platelet activation [262]. Through these mechanisms, vitamin E attenuates platelet aggregation, reduces the oxidative modification of LDL, suppresses vascular inflammation, and limits lipid accumulation within the arterial wall, collectively contributing to the prevention of atherosclerotic plaque formation and progression. Despite substantial experimental and epidemiological evidence supporting the antioxidant, anti-inflammatory, and anti-atherosclerotic properties of vitamin E, clinical trial findings have often produced inconsistent outcomes. While vitamin E effectively inhibits lipid peroxidation and modulates redox-sensitive signaling pathways implicated in CVD, large-scale intervention studies have generally failed to demonstrate significant cardiovascular benefits. Notably, the Heart Outcomes Prevention Evaluation (HOPE) and its extension study (HOPE-TOO), involving patients at high cardiovascular risk, found that long-term supplementation with vitamin E (400 IU/day) did not significantly reduce major cardiovascular events compared with placebo [263]. Furthermore, vitamin E supplementation was associated with a 13% higher risk of heart failure and a 21% higher risk of hospitalization for heart failure [263]. These findings suggest that antioxidant activity alone may be insufficient to improve cardiovascular outcomes and highlight the complexity of redox regulation in human disease. Moreover, evidence from clinical studies and meta-analyses indicates that high-dose vitamin E supplementation should not be assumed to be universally safe, as beneficial effects appear to depend on factors such as dosage, baseline nutritional status, disease condition, and individual patient characteristics. In particular, a meta-analysis of 19 randomized controlled trials involving 135,967 participants reported that high-dose vitamin E supplementation (≥400 IU/day) may be associated with a small but statistically significant increase in all-cause mortality, suggesting the need for caution when considering long-term high-dose supplementation [264].

Beyond its established roles in cardiovascular and neuroprotective health, vitamin E isoforms, particularly tocotrienols, have also been investigated for potential anticancer effects. Accumulating evidence indicates that tocotrienols exert antitumor effects through the modulation of multiple signaling pathways involved in cell proliferation, survival, and metastasis. For example, annatto-derived tocotrienol (20 μg/mL for 24 h) significantly inhibited the proliferation of PC-3 prostate cancer cells by suppressing the activation of signal transducer and activator of transcription 3 (STAT3) and Src kinase, two oncogenic signaling molecules that regulate tumor growth and survival [265]. Consistent with these findings, Tan and Norhaizan [266] reported that antioxidant-rich dietary components may contribute to the prevention of prostate carcinogenesis by attenuating oxidative stress, a key driver of DNA damage, genomic instability, and tumor initiation. Furthermore, a dose–response meta-analysis demonstrated that lung cancer risk decreased by approximately 5% for every 2 mg/day increase in dietary vitamin E intake, suggesting a potential protective role of vitamin E against carcinogenesis [172]. Vitamin E regulates apoptosis through the activation of intrinsic and extrinsic cell death pathways, suppresses cancer cell invasion by modulating MMPs and epithelial–mesenchymal transition-related signaling, and enhances antitumor immunity by influencing immune cell function within the tumor microenvironment [267].

### 6.8. Ubiquinone

Coenzyme Q10 (CoQ10), also known as ubiquinone, is an endogenously synthesized lipid-soluble quinone that functions as both a component of the mitochondrial ETC and a potent antioxidant within biological membranes [268]. Structurally, CoQ10 consists of a benzoquinone ring linked to a hydrophobic isoprenoid side chain, enabling its localization within cellular membranes, particularly the inner mitochondrial membrane, where it facilitates electron transfer between complexes I/II and complex III during oxidative phosphorylation. Since its first isolation from bovine heart mitochondria in 1957, CoQ10 has gained considerable attention due to its essential role in cellular bioenergetics and redox homeostasis. Given its involvement in ATP production, CoQ10 is highly concentrated in metabolically active tissues with elevated energy demands, including the heart, liver, kidney, and brain [269]. Although endogenous biosynthesis represents the primary source of CoQ10, dietary intake can contribute to systemic levels, with meat, fish, and other animal-derived products serving as the richest dietary sources [270].

CoQ10 is a potent endogenous lipophilic antioxidant that plays a role in mitochondrial energy metabolism by serving as an electron carrier within the ETC during oxidative phosphorylation [271]. Through its reversible conversion between oxidized (ubiquinone) and reduced (ubiquinol) forms, CoQ10 participates in cellular redox cycling and effectively neutralizes ROS, thereby protecting membrane phospholipids, plasma lipoproteins, and mitochondrial components from oxidative damage [272]. These antioxidant properties are particularly important in the cardiovascular system, where oxidative stress contributes to endothelial dysfunction and atherogenesis. CoQ10 has been suggested to exert cardioprotective effects by inhibiting the oxidative modification of LDL cholesterol, thereby reducing lipid peroxidation and the formation of oxidized LDL, a key initiator of vascular inflammation and atherosclerotic plaque development [192].

Emerging evidence indicates that CoQ10 status is closely linked to metabolic health, as higher circulating CoQ10 concentrations have been associated with a lower risk of metabolic syndrome and elevated HDL-C levels [273]. These associations may be attributed to the ability of CoQ10 to preserve mitochondrial function and regulate cellular redox homeostasis, both of which are frequently impaired in metabolic disorders. By enhancing electron transport efficiency and reducing mitochondrial ROS generation, CoQ10 may alleviate oxidative stress-induced insulin resistance, dyslipidemia, and chronic low-grade inflammation. In addition, CoQ10 has been suggested to modulate key metabolic pathways governing lipid utilization and cholesterol transport, which may contribute to improved HDL-mediated reverse cholesterol transport and overall lipid homeostasis.

The effects of CoQ10 on oxidative stress and inflammatory status have been extensively investigated in patients with CAD. In a systematic review and meta-analysis of placebo-controlled, double-blind randomized controlled trials involving 364 participants in the intervention groups and 349 participants in the control groups, supplementation with CoQ10 at doses ranging from 60 to 300 mg/day for 4–48 weeks significantly reduced concentrations of conjugated dienes and malondialdehyde (MDA), while increasing the activities of endogenous antioxidant enzymes, including catalase and SOD [186]. These findings suggest that CoQ10 enhances antioxidant defense mechanisms and attenuates lipid peroxidation, thereby mitigating oxidative stress, a key contributor to endothelial dysfunction and atherosclerotic progression in CAD. However, despite these improvements in oxidative stress biomarkers, CoQ10 supplementation did not significantly affect GPx activity or circulating levels of inflammatory markers, including IL-6, TNF-α, and CRP [186], implying the complexity of the relationship between redox modulation and disease outcomes. These findings suggest that reductions in oxidative stress biomarkers alone may be insufficient to achieve clinical benefits. Collectively, the observed changes in oxidative stress biomarkers suggest that CoQ10 may contribute to the maintenance of redox homeostasis.

### 6.9. Organosulfur Compounds

Organosulfur compounds constitute a diverse class of bioactive phytochemicals that are particularly abundant in vegetables of the *Brassica* and *Allium* genera, such as broccoli, cabbage, cauliflower, garlic, and onion [274]. These compounds contribute significantly to the characteristic sensory attributes and health-promoting properties of *Allium* vegetables. Volatile lipid-soluble sulfur compounds are primarily responsible for the pungent aroma and flavor of garlic and onion, whereas water-soluble derivatives, including *S*-allyl cysteine (SAC) and *S*-allyl mercaptocysteine (SAMC), are major constituents of aged or aqueous garlic extracts and possess notable biological activities [275]. Upon tissue disruption, enzymatic conversion of sulfur-containing precursors generates an extensive range of reactive organosulfur metabolites that contribute to both flavor development and bioactivity [276]. In onions, sulfur-containing intermediates, including mono-, di-, and trisulfides, thiosulfinates, and thiosulfonates, have been identified as key contributors to their characteristic flavor profile [277]. Accumulating evidence further indicates that dietary organosulfur compounds, such as propyl disulfide, dimethyl disulfide, and diallyl disulfide, exert vasodilatory and anti-inflammatory effects and may confer protection against the development of chronic diseases [278]. Glucosinolates are naturally occurring sulfur-rich phytochemicals characteristic of *Brassica* vegetables and constitute the primary precursors of isothiocyanates, a group of bioactive metabolites extensively studied for their antioxidant, anti-inflammatory, and chemopreventive properties [279].

Organosulfur compounds have attracted considerable scientific interest because of their diverse biological activities and potential role in disease prevention. Accumulating evidence indicates that these compounds exert anticancer effects by targeting multiple molecular pathways involved in tumor initiation, promotion, and progression [280]. Mechanistic studies have demonstrated that organosulfur compounds derived from *Allium* vegetables regulate carcinogen metabolism through induction of phase II detoxification enzymes, inhibition of carcinogen activation, attenuation of oxidative stress, modulation of cell-cycle checkpoints, activation of apoptosis, and maintenance of redox homeostasis [281]. Such multitargeted actions position organosulfur compounds as a dietary chemopreventive agents.

Consistent with mechanistic findings, epidemiological studies have associated higher consumption of garlic, onions, and broccoli sprouts with a lower incidence of several cancers, particularly breast, prostate, and colorectal cancers [282]. A meta-analysis involving 12,558 cases further reported a significant inverse association between garlic consumption and colorectal cancer risk [283]. Notably, among the various phytochemicals present in *Allium* vegetables, organosulfur compounds appear to contribute substantially to their protective effects against both cancer and metabolic disorders [284]. In garlic, aged garlic extract is a rich source of the water-soluble organosulfur compounds SAC and SAMC, which exhibit antioxidant and anticancer activities [285]. For example, SAC has been shown to induce apoptosis in neuroblastoma cells via mitochondrial permeability transition-dependent mechanisms, highlighting a plausible molecular basis for the anticancer effects of garlic-derived organosulfur compounds [286].

The anticarcinogenic properties of garlic-derived organosulfur compounds are mediated through multiple mechanisms, including the attenuation of oxidative stress and modulation of epigenetic signaling pathways. For example, SAC has been shown to induce apoptosis in neuroblastoma cells while simultaneously protecting cellular macromolecules against oxidative damage by suppressing protein oxidation and lipid peroxidation [286]. Consistent with these findings, in vivo studies have demonstrated that long-term dietary supplementation with broccoli sprouts or sulforaphane significantly reduces prostate cancer incidence in mice, an effect associated with the downregulation of histone deacetylase 3 (HDAC3) expression in prostate epithelial tissue [287]. These observations suggest that organosulfur compounds may exert chemopreventive effects through a combination of antioxidant, pro-apoptotic, and epigenetic regulatory mechanisms. Sulforaphane, a bioactive isothiocyanate derived from glucosinolate hydrolysis in cruciferous vegetables, has emerged as one of the most extensively studied organosulfur compounds owing to its potent antioxidant and anti-inflammatory activities [288]. Mechanistically, sulforaphane exerts its anti-inflammatory effects through activation of the Nrf2/HO-1 signaling pathway, a regulator of cellular redox homeostasis. In LPS-induced RAW 264.7 macrophages, sulforaphane (10–20 μM) markedly suppressed NO production and downregulated the expression of iNOS and proinflammatory cytokines, thereby attenuating inflammatory responses [289]. These findings suggest that modulation of Nrf2-dependent cytoprotective pathways represents a major mechanism through which sulforaphane confers its beneficial biological effects.

Although substantial evidence supports the anticancer potential of sulforaphane, findings from human studies remain inconsistent. Experimental studies have demonstrated that sulforaphane-rich broccoli sprouts exert chemopreventive effects through the modulation of pathways governing epigenetic regulation, apoptosis, cell-cycle control, DNA repair, and transcriptional activity [290]. Translational evidence from a 12-month intervention study further indicated that regular consumption of glucoraphanin-rich broccoli soup may reduce the likelihood of prostate cancer progression by altering transcriptional responses within prostate tissue [180]. Nevertheless, these observations were not replicated in clinical settings. For example, supplementation with 200 μmol/day sulforaphane-rich broccoli sprout extract for 20 weeks failed to induce clinically meaningful reductions in serum prostate-specific antigen (PSA) levels among men with recurrent prostate cancer [179]. Given that elevated PSA concentrations are strongly associated with prostate cancer progression and disease burden [291], the absence of significant PSA responses suggests that the clinical benefits of sulforaphane may be context-dependent. Collectively, the available evidence highlights the need for further investigation into the optimal dosage, duration, formulation, and target population for sulforaphane-based interventions.

In addition to their established anticancer and anti-inflammatory activities, organosulfur compounds are increasingly recognized for their role in cardiovascular protection. Clinical evidence indicates that garlic and aged garlic extract supplementation (400–2400 mg/day) can improve vascular function, particularly in populations with elevated cardiovascular risk [182]. The cardiovascular benefits of garlic are largely attributed to its complex organosulfur composition, which undergoes substantial biochemical transformation during processing and consumption. Fresh garlic contains γ-glutamylcysteines that serve as precursors of alliin, which is enzymatically converted by alliinase into allicin upon tissue disruption [292]. Because allicin is highly reactive and unstable, it rapidly decomposes into numerous secondary organosulfur compounds, including ajoene and diallyl trisulfide (DATS), that are responsible for many of garlic’s biological effects.

DATS has emerged as a particularly promising bioactive metabolite owing to its antiplatelet and lipid-modulating properties. Experimental studies have shown that DATS suppresses platelet aggregation through regulation of sulfhydryl-mediated reactions, thereby potentially reducing thrombotic risk [293]. Furthermore, DATS supplementation significantly improved lipid metabolism in rats with metabolic syndrome, as evidenced by increased HDL-C levels and reductions in LDL-C and triglycerides [124]. The underlying mechanism has been linked to inhibition of endogenous cholesterol synthesis [294]. Emerging evidence indicates that DATS may provide cardiovascular protection through its ability to modulate key molecular pathways involved in myocardial injury. In a metabolic syndrome rat model, DATS significantly alleviated ex vivo ischemia–reperfusion-induced cardiac damage by reducing oxidative stress, inflammatory responses, and apoptosis in myocardial tissue [124]. Given that oxidative stress and chronic inflammation play central roles in the pathogenesis of both CVD and metabolic dysfunction, the ability of DATS to simultaneously target these processes suggests the therapeutic role of this garlic-derived organosulfur compound. These findings further support the notion that organosulfur compounds may confer cardiovascular benefits beyond lipid regulation, extending to the prevention of cardiac injury and other metabolic syndrome-related complications.

The metabolic benefits of organosulfur compounds extend beyond their established anticancer and cardioprotective activities to include potential improvements in glucose regulation and insulin sensitivity [295]. Since progressive β-cell dysfunction and insulin resistance are central events in the pathogenesis of T2DM, dietary compounds capable of modulating these processes have attracted substantial interest [296]. Observational evidence indicates an inverse association between the consumption of organosulfur-rich foods and the risk of diabetes [297].

A longitudinal investigation by Mirmiran et al. [200] demonstrated that individuals with the highest consumption of raw onion and garlic (≥142 g/week) had a significantly lower likelihood of developing hyperinsulinemia and insulin resistance than those with minimal intake (<8 g/week). Although *Allium* vegetable consumption was not associated with improved β-cell function, the observed reduction in insulin resistance suggests that organosulfur compounds may contribute to the maintenance of glucose homeostasis through peripheral metabolic mechanisms. Mechanistically, these effects may be partly attributable to their antioxidant activity. Increased oxidative stress is a hallmark of diabetes and has been linked to impaired insulin signaling and β-cell dysfunction. In support of this relationship, diabetic rats exhibit increased DNA damage and diminished antioxidant defense capacity, characterized by lower total antioxidant capacity and GSH levels [298]. Therefore, the ability of organosulfur compounds to attenuate oxidative stress and restore redox balance may represent a key mechanism mediating their antidiabetic activity.

### 6.10. Ergothioneine

Ergothioneine has emerged as a dietary antioxidant because of its stability and ability to accumulate in tissues exposed to high levels of oxidative stress. This naturally occurring sulfur-containing amino acid is synthesized by selected fungi, cyanobacteria, and actinobacteria and is subsequently acquired by humans through dietary consumption [299], with edible mushrooms representing the richest dietary sources. Oyster mushrooms (*Pleurotus* spp.) and boletes (*Boletus* spp.) contain particularly high concentrations in ergothioneine [300,301]. Chemically, ergothioneine is a histidine-derived thiourea compound containing a sulfur atom within the imidazole ring, a structural feature that contributes to its remarkable redox properties [299]. As a potent scavenger of •OH and other ROS, ergothioneine protects cellular biomolecules from oxidative damage and helps maintain redox homeostasis [302]. Growing evidence further indicates that ergothioneine may exert protective effects against a range of chronic diseases and environmental stressors, including ultraviolet radiation-induced cellular injury, highlighting its potential as a multifunctional cytoprotective compound [299,303]. Consequently, regular mushroom consumption of ergothioneine-rich foods has been proposed as a practical dietary strategy to increase dietary intake of this bioactive compound and support antioxidant defense mechanisms.

Unlike most dietary antioxidants, ergothioneine exhibits a unique biological disposition characterized by selective uptake and tissue accumulation. Although biosynthesis is restricted to certain fungi, actinobacteria, and cyanobacteria, humans acquire ergothioneine primarily through dietary sources, particularly mushrooms, legumes, and animal-derived foods such as liver and kidney [299,304]. Cellular uptake of ergothioneine is mediated by the highly specific transporter organic cation transporter novel 1 (OCTN1) (solute carrier family 22 member 4 (SLC22A4)), reflecting its physiological importance despite the inability of the molecule to passively cross plasma membranes [305]. Notably, ergothioneine accumulates in tissues vulnerable to oxidative and inflammatory injury, including the liver and kidneys, suggesting an adaptive role in protecting cells from oxidative damage and maintaining redox balance. Growing evidence indicates that ergothioneine may play a protective role in cardiovascular health and longevity. A large prospective cohort study comprising 3236 individuals without pre-existing stroke, CAD, or type 2 diabetes reported that higher ergothioneine concentrations were significantly associated with lower risks of all-cause mortality, cardiovascular mortality, and CAD during 21.4 years of follow-up [183]. Because oxidative stress is recognized as a key driver of endothelial dysfunction, atherosclerosis, and CVD progression [306], the capacity of ergothioneine to scavenge ROS and maintain redox homeostasis may partly explain its favorable association with cardiovascular outcomes.

### 6.11. Betalains

Betalains are water-soluble, nitrogen-containing pigments composed of two structurally distinct subclasses, namely, betacyanins and betaxanthins, which are responsible for red-violet and yellow-orange pigmentation, respectively [307]. All betalain molecules originate from a common betalamic acid backbone, while structural modifications generate the diverse range of naturally occurring betalain derivatives. Representative betacyanins, including betanin, isobetanin, prebetanin, and neobetanin, exhibit absorption maxima around 538 nm, whereas betaxanthins absorb at approximately 480 nm [307]. Beyond their role as natural pigments, betalains have been demonstrated to exert multiple biological activities, including antioxidant, anti-inflammatory, antibacterial, hepatoprotective, immunomodulatory, and anticarcinogenic effects. Furthermore, growing evidence indicates that these compounds contribute to the regulation of intestinal homeostasis and immune function [308].

Betalains possess antioxidant activity and are highly effective in scavenging a variety of reactive oxygen and nitrogen species [309]. The potent antioxidant capacity of betalains is largely attributed to their unique molecular structure, which facilitates electron or hydrogen atom donation to neutralize free radicals. Specifically, the presence of conjugated double bonds, aromatic rings, and electron-rich functional groups enables betalains to effectively quench reactive species and interrupt free radical chain reactions [307]. Among the betalain subclasses, betacyanins generally exhibit stronger antioxidant activity than betaxanthins [310]. Consistent with this observation, both betacyanins and betaxanthins isolated from red beetroot have demonstrated potent antiradical activity against 2,2′-azino-bis(3-ethylbenzothiazoline-6-sulfonic acid) (ABTS) radicals [311]. Through their ability to modulate cellular redox balance and inhibit LDL oxidation, betalains may contribute to the prevention of oxidative stress-related disorders and promote a favorable intracellular redox environment [312].

Beyond their antioxidant properties, betalains also exhibit significant anti-inflammatory activity through multiple complementary mechanisms. Experimental studies have demonstrated that betalains suppress the production of reactive oxidants, scavenge HOCl, and inhibit COX-mediated inflammatory responses generated by activated neutrophils [312]. The close interplay between oxidative stress and inflammation further supports the therapeutic potential of betalains, as excessive oxidative stress can activate NF-κB signaling, thereby promoting the expression of proinflammatory mediators [313,314,315]. Consequently, the antioxidant activity of betalains may indirectly attenuate inflammatory responses through the modulation of redox-sensitive signaling pathways.

Human studies also provide evidence supporting the antioxidant effects of dietary betalains. Tesoriere et al. [316] detected betanin in erythrocytes of healthy individuals within three hours of consuming a cactus pear fruit meal, indicating that betalains are bioavailable and can be incorporated into circulating blood cells. Notably, erythrocytes collected after betalain consumption exhibited significantly delayed ex vivo cumene hydroperoxide-induced hemolysis, suggesting enhanced resistance to oxidative damage. These findings collectively indicate that betalains exert potent antioxidant and anti-inflammatory effects through both direct free radical scavenging and modulation of redox-sensitive inflammatory pathways, highlighting their potential role in the prevention and management of chronic diseases associated with oxidative stress and inflammation.

Growing evidence supports the role of betalains in mitigating cardiovascular and metabolic disorders. In animal models of myocardial injury, betalain-rich beetroot preparations have demonstrated cardioprotective effects by restoring endogenous antioxidant systems and suppressing oxidative and nitrosative stress [127]. Notably, these effects were associated with reduced tissue injury, decreased inflammatory damage, and modulation of apoptosis-related proteins, including enhanced B-cell lymphoma 2 (Bcl-2) expression and reduced MMP-9 levels. Such findings suggest that betalains may preserve myocardial integrity through coordinated regulation of oxidative stress, inflammatory signaling, and cell survival pathways.

In addition to their direct cardioprotective effects, betalains may also improve metabolic risk factors that contribute to CVD. Dietary administration of betalain-rich beetroot crisps significantly lowered hepatic cholesterol and circulating glucose levels in hyperlipidemic mice [130], indicating potential benefits in the management of dyslipidemia and impaired glucose metabolism. Given the close relationship between metabolic abnormalities, oxidative stress, and CVD progression, these findings support the hypothesis that betalains may exert broad cardiometabolic protective effects through multiple complementary mechanisms.

Emerging evidence suggests that dietary intake of betalain-rich foods may contribute to cancer prevention through their antioxidant, anti-inflammatory, and cytoprotective activities. Among the proposed mechanisms, activation of the Nrf2 signaling pathway appears to play a central role. Betalains derived from red beetroot have been shown to activate Nrf2 and induce the expression of phase II detoxification enzymes, thereby enhancing endogenous antioxidant defense systems and cellular protection against oxidative stress [312].

Experimental studies further support the chemopreventive potential of betalains. Krajka-Kuźniak et al. [317] reported that dietary supplementation with red beetroot juice (8 mL/kg body weight/day) for 28 days significantly attenuated N-nitrosodiethylamine (NDEA)-induced DNA damage and liver injury in rats. Consistent with these findings, betalains at concentrations of 2, 10, and 20 μM activated Nrf2 signaling and upregulated downstream phase II detoxification genes in normal human liver (THLE-2) cells, suggesting a potential role in both hepatoprotection and carcinogen detoxification [317]. These observations indicate that betalains may exert anticarcinogenic effects by enhancing cellular antioxidant defenses and promoting the elimination of potentially harmful compounds.

In addition to their chemopreventive properties, betalains have demonstrated direct anticancer activity in vitro. Farabegoli et al. [318] showed that red beetroot-derived betalains induced cytotoxicity in Caco-2 colon cancer cells through the activation of apoptotic pathways, as evidenced by increased expression of Bcl-2-like protein 4 (Bax), cleaved caspase-3, and poly(ADP-ribose) polymerase-1 (PARP-1). Furthermore, betalain treatment reduced the expression of proinflammatory mediators, including interleukin-8 (IL-8) and COX-2, in lipopolysaccharide-stimulated Caco-2 cells. These findings suggest that betalains may suppress tumor progression through the coordinated modulation of apoptosis, inflammation, and oxidative stress pathways. Collectively, the available evidence highlights the potential of betalains as multifunctional phytochemicals capable of mitigating oxidative stress, metabolic dysfunction, inflammation, and cancer development. Nevertheless, it is important to note that most studies to date have evaluated whole red beetroot extracts rather than isolated betalain compounds. Because red beetroot also contains other bioactive constituents, including betaine, ferulic acid, and lutein, the specific contribution of individual betalains to the observed health benefits remains difficult to ascertain [319]. Therefore, further mechanistic, translational, and clinical studies are warranted to elucidate the bioavailability, molecular targets, and therapeutic efficacy of individual betalain compounds within the context of habitual betalain-rich diets.

### 6.12. Carnosine and Anserine

Carnosine is a naturally occurring histidine-containing dipeptide composed of β-alanine and L-histidine and is widely distributed in excitable tissues such as skeletal muscle, cardiac muscle, and the brain. Structurally, carnosine contains three ionizable groups, namely, the histidine imidazole ring, the β-alanine amino group, and the terminal carboxyl group, which collectively confer unique buffering, metal-chelating, and antioxidant properties [320]. Under physiological pH conditions, carnosine predominantly exists as a zwitterion, enabling efficient interactions with reactive species and cellular macromolecules [321]. Dietary carnosine is absorbed in the small intestine through PepT1-mediated transport and is partially hydrolyzed by intracellular carnosinase-2 within enterocytes [322]. Following absorption, circulating carnosine can be degraded by serum carnosinase-1 into histidine and β-alanine, which are subsequently utilized for tissue uptake and intracellular carnosine resynthesis. Importantly, dietary intake of carnosine has been shown to elevate tissue carnosine concentrations, particularly in skeletal muscle, cardiac tissue, and the central nervous system, suggesting a role in maintaining cellular homeostasis and protecting against oxidative and metabolic stress [323].

The biological activities of carnosine are largely attributed to its functional imidazole ring, which readily donates electrons or hydrogen atoms to neutralize ROS and free radicals, thereby limiting oxidative damage to cellular components [324]. Owing to its unique chemical structure, carnosine exerts multiple physiological functions across various tissues and organs. These include protection against protein oxidation, lipid peroxidation, AGEs, and advanced lipoxidation end products (ALEs), all of which are implicated in aging and chronic disease pathogenesis. In addition, carnosine acts as an efficient scavenger of ROO• and other ROS, contributes to metal-ion chelation, and regulates cellular redox homeostasis. Beyond its antioxidant properties, carnosine facilitates skeletal muscle function through the modulation of ATPase activity and serves as an important intracellular pH buffer, particularly in metabolically active tissues such as skeletal and cardiac muscle [325]. Collectively, these multifunctional properties position carnosine as an endogenous cytoprotective molecule involved in maintaining cellular integrity under conditions of oxidative and metabolic stress.

Following the discovery of carnosine in beef, subsequent investigations sought to identify related histidine-containing dipeptides in other animal species. In 1929, Ackermann et al. [326] and Tolkatschevskaya [327] independently reported the presence of a carnosine-like compound, β-alanyl-1-methyl-L-histidine, in goose skeletal muscle. This peptide was later designated anserine, the taxonomic genus of goose. Structurally, anserine contains three ionizable functional groups, namely, the imidazole ring of histidine, the amino group of the β-alanine residue, and the carboxyl group, which contribute to its physicochemical properties [328]. Under physiological conditions, anserine predominantly exists in a zwitterionic form with an overall positive charge [329]. Dietary anserine is mainly obtained from animal-derived foods, particularly beef and fish species such as trout, tuna, and salmon [330,331]. Unlike carnosine, anserine is not detected in human tissues, including the brain, heart, and skeletal muscle [332]. Anserine shares several physiological activities with carnosine, including antioxidant and cytoprotective properties [330,333,334]. Nevertheless, notable biochemical differences exist between these dipeptides. For example, anserine exhibits limited copper-chelating capacity and may therefore exert a lesser influence on cellular NO bioavailability compared with carnosine [330].

Both anserine and carnosine are histidine-containing dipeptides with potent antioxidant properties that contribute to cellular protection against oxidative damage [335]. Accumulating evidence indicates that these bioactive peptides play important roles in mitigating oxidative stress and preserving cellular integrity under pathological conditions [336,337]. In a diabetic nephropathy model, intravenous administration of anserine (100 mg/kg, three doses administered every other day) to male C57BL/KsJm/Leptdb (db/db) mice significantly reduced blood glucose levels and proteinuria while improving vascular permeability, suggesting a protective effect against diabetes-associated vascular and renal dysfunction [128]. These findings are particularly relevant given that impaired stress-response mechanisms are a hallmark of diabetic complications, where reduced expression of heat shock protein 70 (Hsp70) has been linked to increased susceptibility to renal injury and disease progression [338,339]. Collectively, these findings suggest that carnosine and anserine may attenuate oxidative stress-related cellular dysfunction and represent a nutritional or therapeutic interventions for obesity-associated diseases. However, current evidence is derived predominantly from experimental and preclinical studies, whereas data from human intervention trials remain limited. Consequently, well-designed randomized controlled trials are required to determine whether long-term consumption of carnosine- and anserine-rich foods can effectively modulate mitochondrial oxidative stress and improve clinical outcomes in chronic diseases associated with obesity.

## 7. Limitation of the Study

While numerous in vitro and in vivo studies have demonstrated the antioxidant, anti-inflammatory, and metabolic benefits of dietary phytochemicals, their translation into clinical efficacy remains challenging due to limitations in bioavailability and pharmacokinetics. Following ingestion, many antioxidant compounds undergo extensive digestion, intestinal absorption, first-pass metabolism, and phase II conjugation reactions, resulting in circulating metabolites that differ substantially from the parent compounds commonly evaluated in experimental models [340,341]. Moreover, gut microbiota-mediated biotransformation further modifies the chemical structures and biological activities of many dietary antioxidants before they enter systemic circulation [341]. Consequently, the bioactive species present in vivo are often glucuronidated, sulfated, or methylated metabolites rather than the unconjugated forms used in cell culture experiments.

Many in vitro experiments employ concentrations that may not be physiologically achievable through normal dietary intake. For example, phytochemicals such as polyphenols, flavonoids, carotenoids, and organosulfur compounds are frequently tested at concentrations ranging from 10 to 100 μM in cultured cells, whereas circulating concentrations following consumption of fruits, vegetables, or other dietary sources are often substantially lower and may exist predominantly as conjugated metabolites [340]. Thus, a compound demonstrating potent antioxidant or anti-inflammatory activity in RAW264.7 macrophages or other cellular models may not reach comparable free concentrations in human tissues after consumption of antioxidant-rich foods such as broccoli, berries, or cruciferous vegetables. Furthermore, absorption efficiency, food matrix interactions, interindividual variability, genetic factors, disease status, and differences in gut microbiota composition can significantly influence systemic exposure and therapeutic responsiveness [341,342]. Differences between naturally occurring dietary antioxidants consumed as part of whole foods and isolated antioxidant supplements further complicate interpretation of the evidence. Whole foods contain complex mixtures of bioactive compounds that may act synergistically, whereas supplementation with high doses of isolated antioxidants may not reproduce these effects and, in some circumstances, may even disrupt physiological redox balance. Importantly, the biological significance of ROS extends beyond their role as mediators of oxidative damage. Contemporary redox biology recognizes ROS as indispensable signaling molecules that regulate cellular adaptation, metabolism, immune responses, and stress signaling. Therefore, the therapeutic potential of antioxidants should not be interpreted solely in terms of ROS scavenging. Rather, their biological effects appear to involve the modulation of redox-sensitive signaling networks and restore redox homeostasis under conditions of oxidative distress, thereby contributing to the maintenance or restoration of redox homeostasis under pathological conditions [343].

## 8. Conclusions

Obesity-induced redox dysregulation represents a key pathogenic mechanism underlying the progression of metabolic, cardiovascular, and cancer-related complications. Excessive generation of ROS and impaired antioxidant defenses promote chronic inflammation, lipid peroxidation, mitochondrial dysfunction, and aberrant cellular signaling, thereby exacerbating obesity-associated disease burden. Antioxidants may mitigate oxidative damage and restore redox homeostasis by targeting multiple pathways involved in obesity-related disease progression. By reducing ROS levels, antioxidants may prevent oxidative DNA damage and tumor initiation; however, excessive suppression of ROS may also promote cancer cell survival and interfere with the efficacy of certain ROS-dependent anticancer therapies. Despite the substantial mechanistic and preclinical evidence supporting their beneficial effects, clinical findings regarding the efficacy of antioxidant interventions remain inconsistent and inconclusive. The variability in outcomes may stem from methodological limitations, including insufficient study duration, inadequate sample size, differences in baseline antioxidant status, and the complex interactions between antioxidants and endogenous defense systems. While oxidative stress is widely implicated in the pathogenesis of obesity-induced diseases, reducing ROS is not invariably beneficial. ROS are not solely harmful by-products of metabolism but also function as essential signaling molecules involved in cell proliferation, differentiation, immune defense, mitochondrial adaptation, and metabolic regulation. Physiological ROS levels are required to maintain cellular redox homeostasis and regulate multiple signaling pathways, whereas oxidative damage occurs when ROS production exceeds antioxidant defense capacity. Consequently, the indiscriminate suppression of ROS may interfere with normal physiological processes and adaptive stress responses. This concept may partly explain why antioxidant interventions that demonstrate promising results in experimental models frequently fail to produce consistent clinical benefits. Other factors, including poor bioavailability, insufficient tissue targeting, interindividual variability, disease heterogeneity, inappropriate timing of intervention, and differences between experimental and clinical settings, further contribute to inconsistent outcomes in human studies. Therefore, future therapeutic strategies should focus not only on ROS scavenging but also on restoring redox balance while preserving physiologically important redox signaling pathways. Importantly, recent research has shifted from evaluating individual antioxidants toward examining dietary patterns and whole-food approaches. This paradigm shift is based on emerging evidence that multiple antioxidants and phytochemicals may act synergistically, enhancing their collective capacity to modulate redox homeostasis, inflammation, and cellular signaling pathways. Such synergistic effects suggest that the benefits of antioxidant-rich diets are unlikely to be attributable to a single compound but rather to the integrated actions of diverse bioactive constituents. Therefore, although antioxidants are unlikely to function as pharmacological cures, they remain a modulator of oxidative stress and obesity-associated disease prevention. Future long-term, randomized controlled trials with robust methodological designs are essential to establish causal relationships between antioxidant-rich dietary interventions and the reduction in oxidative stress and obesity-induced diseases.

## Figures and Tables

**Table 1:** In vitro and in vivo studies conducted in several antioxidants and their effects in obesity-associated diseases.

Obesity-Associated Diseases	Animal Study/Cell Lines	Antioxidants	Dosages/Duration	Findings	References
Colon and breast cancers	LoVo and MCF-7 cell lines	Peel flavonoids (Peel-F) and flesh flavonoids (Flesh-F) extracted from Pink Lady apples	LoVo cells (Peel-F = 110.33 ± 2.52 mg/mL and Flesh-F = 378.14 ± 1.64 mg/mL)	↓ Proliferation of LoVo and MCF-7 cells	[116]
			MCF-7 cells (Peel-F = 58.42 ± 1.39 mg/mL and Flesh-F = 296.06 ± 3.71 mg/mL)		
Colon cancer	Pirc rats (F344/ NTac-Apc^am1137^)	Flavonoid-rich extract from bergamot juice	35 mg/kg and 70 mg/kg body weight for 12 weeks	↓ Colon preneoplastic lesions mucin-depleted foci	[117]
Hepatocellular carcinoma	Systematic review and meta-analysis involving 644 animals (Long Evans Cinnamon rats, ferrets, C57BL/6J mice, C3H/HeN mice, WT/BCO2-KO mice, Sprague Dawley rats, Wistar rats, and BALB/c mice), studies between 2001 and 2018	Lycopene	1.1–15 mg/kg/day or 20 mg/kg for 3 times a week, range from 4 to 70 weeks via diet	↓ Growth, number, and incidence of hepatocellular carcinoma	[118]
Colon cancer	Colon cancer cells (C2BBe1)	Ascorbic acid supplements	0.95 mM for 24 h	↓ Cell proliferation	[119]
Liver cancer	Rats	Red beetroot juice	8 mL/kg body weight/day for 28 days	Decreased the NDEA-induced DNA damage and liver injury	[120]
Cardiovascular disease	Male Wistar rats	(−)-epigallocatechin-3-gallate	10 mg/kg intravenously	↓ Myocardial damage by reducing NF-κB activity and AP-1 pathway	[121]
Cardiovascular disease	Sprague-Dawley rats	Quercetin	5 μM for 30 min	↓ Vasoconstrictor sensitivity in rat aortic ring	[122]
Cardiovascular disease	Male Brow Norway (BN) rats (*n* = 16) and male Lewis rats (*n* = 32)	Lycopene	30 mg/kg/day for 8 weeks	Prevents transplant vasculopathy	[123]
Cardiovascular disease	36 male Wistar albino rats, the metabolic syndrome rats were fed with high-fat diet for 4 weeks followed by intraperitoneal single streptozotocin injection in a dose of 25 mg/kg	Diallyl trisulfide derived from garlic	40 mg/kg every second day for 3 weeks	Increased HDL-C and reduced LDL-C and TG levels	[124]
Atherosclerosis	Apolipoprotein E-null mice	Carnosine	60 mg/kg in drinking water for 6 weeks	Reduced atherosclerotic lesion formation in aortic valves	[125]
Diabetes mellitus	Systematic review and meta-analysis of animal studies	Quercetin	10, 25, and 50 mg/kg	↓ Serum glucose levels	[126]
Myocardial ischemia	Rats	Betalain-rich beetroot juice	150 and 300 mg/kg for 28 days	Restored cardiac endogenous antioxidants and decreased nitrosative and oxidative stress	[127]
Type 2 diabetes mellitus	Male C57BL/KsJm/Leptdb (db/db) mice	Anserine	3 doses of 100 mg/kg every other day intravenously	Decreased proteinuria and blood glucose levels and improved vascular permeability	[128]
Type 2 diabetes mellitus	Male Sprague–Dawley rats	Betanin	10, 20 and 40 mg/kg/body weight for 28 days	Upregulated AMPK and SIRT1 and downregulated the NF-κB mRNA expression	[129]
Dyslipidemia	Male Wistar rats aged approximately 4 weeks	3% beetroot crisps rich in betalains	4 weeks	Reduced hepatic total cholesterol and serum glucose levels	[130]

AMPK, adenosine 5′-monophosphate-activated protein kinase; AP-1, activator protein-1; HDL-C, high-density lipoprotein cholesterol; LDL-C, low-density lipoprotein cholesterol; NDEA, N-nitrosodiethylamine; NF-κB, nuclear factor-kappa B; SIRT1, sirtuin-1; TG, triglycerides.

**Table 2:** Clinical studies conducted in several antioxidants and their effects in obesity-associated diseases.

Obesity-Associated Diseases	Population	Antioxidants	Dosages/Duration	Findings	References
Lung cancer	Case–control study (649 incident cases of primary lung cancer among women diagnosed from February 1992 to January 1994 and control group of 675 women)	Polyphenols (Green tea)	>1500 g/year	↓ Lung cancer risk in non-smoking women	[166]
Gastric cancer	European Prospective Investigation into Cancer and Nutrition (EPIC) study (477,312 subjects (29.8% men) aged 35–70 years old from 10 European countries)	Total flavonoid, flavanols, flavones, flavonols, and anthocyanidins	Total flavonoid = (433.8 ± 330.6 mg/day), flavanols (350.8 ± 304.1 mg/day), flavones (3.46 ± 3.92 mg/day), flavonols (26.70 ± 17.38 mg/day), and anthocyanidins (29.51 ± 22.75 mg/day) during an average follow-up of 11 years	↓ Gastric adenocarcinoma risk in women	[167]
Gastric cancer	Case–control study using 334 cases and 334 matched controls aged 35–75 years	Total flavonoid from black soybeans, tofu, and green tea	105.2 ± 77.9 mg/day	↓ Gastric cancer risk in women but not in men	[168]
Gastric cancer	Prospective cohort study (1297 gastric cancer cases)	Flavonoids from 124 food items	≤84.1 mg/day or 84.2–4211.2 mg/day over a mean of 12 years follow-up	No effect	[158]
Lung cancer/prostate cancer	Randomized, double-blind, placebo-controlled primary-prevention trial involving 29,133 male smokers 50–69 years old	Alpha-tocopherol or β-carotene	Alpha-tocopherol (50 mg/day) alone, beta carotene (20 mg/day) alone and follow-up for 5–8 years	No reduction in the incidence of lung cancer among male smokers	[169]
				High incidence of lung cancer in men who received β-carotene compared those who did not received	
				Induce prostate cancer among individuals who received α-tocopherol than those who did not	
Lung cancer	Meta-analysis of epidemiological studies (5 cohort and 12 case–control studies) involving 12,276 cases and 102,516 controls	Coffee	≤1 cup/day, 2–3 cups per day, or ≥3 cups/day	↑ Lung cancer risk	[170]
Lung cancer	Prospective cohort study (63,257 Singaporean Chinese men and women)	Green tea	More than 2 cups/day, with an average of 17.7 years of follow-up	No effect	[171]
Lung cancer	Dose–response meta-analysis involving 9 prospective cohort studies including 431,359 controls and 4164 cases	Dietary vitamin E mainly from soybean oil, soybeans, eggs, and leafy greens	Every 2 mg/day increment	↓ 5% of lung cancer risk	[172]
Cancer	Cohort study (A total of 3234 incident cancer cases among 38,408 women aged 45 years and above)	Foods rich in flavonoids including tofu, onion, broccoli, apple, and tea	Median intake = 8.88–47.44 mg/day during 11.5 years of follow-up	No significant association	[173]
Cancer	Advanced cancer patients	Ascorbic acid supplements	10 g/day	↑ The rate of survival	[174,175]
Pancreatic cancer	Stage IV poorly differentiated pancreatic ductal adenocarcinoma male patient	Ascorbic acid supplements	75–125 g/infusion for 2–3 times/week	Ameliorates the progression of the disease and improved the survival	[176]
Cancer	Different stages of cancers (breast, nasopharynx carcinoma, lung, ovarian, and liver)	Ascorbic acid supplements	25–100 g/day	Improved the quality of life and reduce the risk of metastasis	[177]
Pancreatic cancer	-	Ascorbic acid from diet	22.9–226 mg/day	↓ Risk of pancreatic cancer	[178]
Prostate cancer	Phase II study involving 20 patients with recurrent prostate cancer	Sulforaphane-rich broccoli sprout extracts	200 μmoles/day for 20 weeks	Did not lead to ≥50% PSA declines	[179]
Prostate cancer	Randomized, double-blinded 3-arm parallel intervention study involving 49 localized prostate cancer patients on active surveillance	Glucoraphanin-rich broccoli soup	300 mL/week for 12 months	Inversely related to the risk of prostate cancer progression	[180]
Coronary heart disease	Zutphen Elderly Study (805 males aged 65–84 years)	Flavonoids-containing foods such as apples, onions, or tea	Followed-up for 5 years	↓ Mortality of coronary heart disease	[152]
Cardiovascular disease	Meta-analysis of randomized controlled trials	Catechin supplements	20–300 mg/day	↑ Flow-mediated dilation	[154]
				↓ Pulse wave velocity	
				No changes in endothelial function markers	
Cardiovascular disease	33 male and female patients with coronary artery disease submitted to elective PCI (between 18 and 75 years)	Flavonoid-based antioxidant-rich diet such as grapes, cherry tomatoes, onions, and cruciferous vegetables	40 mg/day antioxidant compounds for 6 months	↓ LDL-C	[181]
				Not related to the decreasing of inflammatory and oxidative stress markers	
Cardiovascular disease	A systematic review of 10 randomized clinical trials between 2004 and 2018	Garlic or aged garlic extract	400–2400 mg/day varied from 15 days to 3 months	Improved vascular function	[182]
Cardiovascular disease	3236 participants free from stroke, coronary artery disease, and type 2 diabetes mellitus aged 57.4 years and follow-up for 21.4 years	Ergothioneine	-	Ergothioneine metabolic level decreased the risk of overall mortality, cardiovascular mortality, and coronary disease	[183]
Cardiovascular disease	Systematic review of 7 human and 9 animal studies	Betalain-rich cacti (cactus pear and dragon fruit)	Animal = varied from 1 to 500 mg/kg body weight between 3 and 60 days; human = varied from 25 mg to 3000 mg between 14 and 56 days	Improved endothelial function, reduced vascular stiffness, and modulated heart rate	[184]
Cardiovascular health	Healthy young adults	Ascorbic acid from fruits and vegetables intakes (including tangerine/orange, kiwi, avocado, papaya, mango, melon, watermelon, nectarine/apricot/peach, grape-fruit, strawberry, pear/apple, banana, pineapple, fig, guava, plum, cherry, cucumber/zucchini/aubergines, peppers, green beans, pumpkin/carrot, tomatoes, cabbage, broccoli/cauliflower, chicory/lettuce, dark-green leaves/spinach, onion, ‘gazpacho’, beetroot, and asparagus	≥150 mg/day	↑ Glutathione peroxidase activity and total antioxidant capacity	[185]
				↓ Plasma ox-LDL levels	
Coronary artery disease	Systematic review and meta-analysis of 13 placebo-controlled and double-blind randomized controlled trials (364 individuals from intervention group and 349 participants in control group)	Ubiquinone supplements	60–300 mg/day of ubiquinone for 4 to 48 weeks	Reduced diene and malondialdehyde concentration, and increased catalase and superoxide dismutase levels	[186]
Coronary artery disease	A randomized, placebo- controlled trial including 51 patients who were identified with cardiac catheterization with at least 50% stenosis of one major coronary artery and having statins for at least 1 month (24 subjects in placebo and 27 participants from ubiquinone group)	Ubiquinone supplement	300 mg/day for 12 weeks	Decreased TNF-α and increased GPx, catalase, and SOD activities	[187]
Cardiovascular disease	Systematic review and meta-analysis including from inception to August 2016, involving 634 participants	Lycopene-containing food such as tomato	70–400 g/day, followed-up for 2 months on average, range from 1 to 180 days	↓ IL-6 levels and LDL-cholesterol	[188]
				Improved flow-mediated dilation	
Cardiovascular disease	Placebo-controlled, randomized, double-blind, cross-over trial with 16 postmenopausal women	100% watermelon juice	Daily dose of 14.4 ± 0.34 mg lycopene for 4 weeks	No effect on serum lipids or antioxidant capacity	[189]
				↑ Circulating lycopene levels	
Cardiovascular disease	Systematic review and meta-analysis of 16 randomized controlled trials and 26 prospective cohort studies	Calcium supplements	1000–1500 mg/day during 6 months-7.1 years of follow-up	↑ Risk of myocardial infarction	[190]
Cardiovascular disease	Systematic review and dose–response meta-analysis of prospective observational studies comprising 7777 cases and 228,531 adults aged 18 years and above	Dietary vitamin E	Every 5 mg/day increment	Not associated with the CVD mortality risk	[191]
Cardiovascular disease	Systematic review and meta-analysis of randomized controlled trials including 12 studies with a total of 650 diabetic patients	Ubiquinone supplement	20–200 mg/day between 8 weeks and 6 months	Reduced LDL-C and total cholesterol level in diabetic patients	[192]
Coronary disease	39,910 U.S. male health professionals aged 40 to 75 years old and follow-up for 4 years	Dietary vitamin E from vegetable oils, nuts, cereal grains, and seeds	Median intake = 12.9 IU/day	↓ Coronary disease risk in men	[193]
Heart failure	Systematic review of 11 randomized controlled trials including 1573 participants	Ubiquinone supplement	Varied from 2 to 10 mg/kg/day in 2 or 3 divided concentration for 6 months to 400 mg/day for 3 months	Reduce the risk of mortality from all causes and hospitalization related to heart failure	[194]
Diabetes mellitus	Meta-analysis involving 8 prospective studies (312,015 subjects with 19,953 developed type 2 diabetes mellitus during the follow-up periods of 4–28 years)	Total flavonoids	≥550 mg/day	↓ Incident of type 2 diabetes mellitus	[195]
Type 2 diabetes mellitus	Phase III, randomized, placebo-controlled study	Selenium supplements	200 μg/day for 33.0 months (range from 0 to 82.6 months)	↑ Risk of type 2 diabetes mellitus in elderly	[196]
Type 2 diabetes mellitus	Systematic review of prospective cohort studies comprising 334,387 participants	Zinc intake (dietary and/or supplements)	Median intake = 4.9–18.0 mg/day and follow-up for 4.8–24 years	No association	[197]
Diabetes mellitus	-	Sour orange juice	240 mL/day for 4 weeks	↓ Fasting blood glucose levels	[198]
Diabetes mellitus	-	Watermelon, bananas, berries (grapes, blueberries, and strawberries), drupes (Japanese apricots, cherries, and peaches), citrus fruits (lemon, grapefruit, and orange), and pome fruits (Japanese persimmons, Japanese pears, and apples), and other melons	22.6–253 g/day	↓ Incidence of diabetic retinopathy	[199]
Type 2 diabetes mellitus	Adults from Tehran Lipid and Glucose Study (2006–2008 to 2009–2011)	*Allium* vegetables (raw onion and garlic)	≥142 g/week	Reduced incidence of hyperinsulinemia and insulin resistance	[200]
Type 2 diabetes mellitus	Type 2 diabetes mellitus patients	Ubiquinone	200 mg CoQ10/day for 12 weeks	Increased nitric oxide production	[201]
Hypertension/diabetes	Hypertensive and/or diabetic obese adults	Ascorbic acid supplements	500 1000 mg/day	No significant effects on several cardiovascular endpoints	[202,203,204]

GPx, glutathione peroxidase; LDL, low-density lipoprotein; ox-LDL, oxidized low-density lipoprotein; PSA, prostate-specific antigen; SOD, superoxide dismutase; TNF-α, tumor necrosis factor-alpha.

## Data Availability

No new data were created or analyzed in this study. Data sharing is not applicable to this article.

## Abbreviations

The following abbreviations are used in this manuscript:

•OH	Hydroxyl radical
^1^O_2_	Singlet oxygen
ABTS	2,2′-azino-bis(3-ethylbenzothiazoline-6-sulfonic acid)
AGE	Advanced glycation end product
AMPK	Adenosine 5′-monophosphate-activated protein kinase
AP-1	Activator protein-1
AREs	Antioxidant response elements
AscH^−^	Ascorbate
AscH_2_	Ascorbic acid
ATP	Adenosine triphosphate
BAT	Brown adipose tissue
Bax	Bcl-2-like protein 4
Bcl-2	B-cell lymphoma 2
CAD	Coronary artery disease
CCR2	CC chemokine receptor 2
cGMP	Cyclic guanosine monophosphate
CoQ10	Coenzyme Q10
COX-2	Cyclooxygenase-2
CRP	C-reactive protein
CVD	Cardiovascular disease
CYP2E1	Cytochrome P450 2E1
DATS	Diallyl trisulfide
DHA	Dehydroascorbic acid
eNOS	Endothelial nitric oxide synthase
ETC	Electron transport chain
FGF-2	Fibroblast growth factor-2
GCL	Glutamate–cysteine ligase
GPx1	Glutathione peroxidase-1
GS	Glutathione synthetase
GSH	Glutathione
GSSG	Glutathione disulfide
GST	Glutathione S-transferase
HDAC3	Histone deacetylase 3
HDL-C	High-density lipoprotein cholesterol
H_2_O_2_	Hydrogen peroxide
HMG-CoA	3-hydroxy-3-methylglutaryl-coenzyme A
HOCl	Hypochlorous acid
IFN-γ	Interferon-gamma
IGF-1	Insulin-like growth factor-1
IL-1	Interleukin-1
IL-1β	Interleukin-1β
IL-6	Interleukin-6
iNOS	Inducible nitric oxide synthase
IRS	Insulin receptor substrates
Keap1	Kelch-like ECH-associated protein 1
LDL	Low-density lipoprotein
LDL-C	Low-density lipoprotein cholesterol
LMWAs	Low-molecular-weight antioxidants
LOX-5	Lipoxygenase-5
MCP-1	Monocyte chemoattractant protein-1
M-CSF	Macrophage colony-stimulating factor
MDA	Malondialdehyde
MMPs	Matrix metalloproteinases
NADPH	Nicotinamide adenine dinucleotide phosphate
NF-κB	Nuclear factor-kappa B
NO	Nitric oxide
Nrf2	Nuclear factor erythroid 2-related factor 2
O_2_•^−^	Superoxide anion
OCl^−^	Hypochlorite
OCTN1	Organic cation transporter novel 1
ox-LDL	Oxidized low-density lipoprotein
PAI-1	Plasminogen activator inhibitor-1
PAR2	Protease-activated receptor 2
PARP-1	Poly(ADP-ribose) polymerase-1
PCSK9	Proprotein convertase subtilisin/kexin type 9
PKC	Protein kinase C
PLA_2_	Phospholipase A_2_
PPP	Pentose phosphate pathway
PSA	Prostate-specific antigen
RNS	Reactive nitrogen species
RO•	Alkoxyl radicals
ROS	Reactive oxygen species
RSS	Reactive sulfur species
SAC	*S*-allyl cysteine
SAMC	*S*-allyl mercaptocysteine
SIRT1	Sirtuin-1
SLC22A4	Solute carrier family 22 member 4
SOD	Superoxide dismutase
STAT3	Signal transducer and activator of transcription 3
T2DM	Type 2 diabetes mellitus
TAMs	Tumor-associated macrophages
TG	Triglycerides
TNF-α	Tumor necrosis factor-alpha
UGT	UDP-glucuronosyltransferase
VCAM-1	Vascular cell adhesion molecule-1
VEGF	Vascular endothelial growth factor
VSMCs	Vascular smooth muscle cells
WAT	White adipose tissue
XO	Xanthine oxidase
